# Synthesis of acyl(chloro)phosphines enabled by phosphinidene transfer†
†Electronic supplementary information (ESI) available. CCDC 1882890–1882892. For ESI and crystallographic data in CIF or other electronic format see DOI: 10.1039/c8sc05657a


**DOI:** 10.1039/c8sc05657a

**Published:** 2019-02-07

**Authors:** Kevin M. Szkop, Michael B. Geeson, Douglas W. Stephan, Christopher C. Cummins

**Affiliations:** a Department of Chemistry , University of Toronto , 80 St George St , Toronto , Ontario M5S3H6 , Canada . Email: dstephan@chem.utoronto.ca ; Tel: +1 416 946 3294; b Department of Chemistry , Massachusetts Institute of Technology , 77 Massachusetts Avenue , Cambridge , MA 02139-4307 , USA . Email: ccummins@mit.edu ; Tel: +1 617 253 5332

## Abstract

Acyl(chloro)phosphines RC(O)P(Cl)(*t*-Bu) have been prepared by formal insertion of *tert*-butyl phosphinidene (*t*-Bu–P) from *t*-BuP**A** (**A** = C_14_H_10_ or anthracene) into the C–Cl bond of acyl chlorides. We show that the under-explored acyl(chloro)phosphine functional group provides an efficient method to prepare bis(acyl)phosphines, which are important precursors to compounds used industrially as radical polymerization initiators. Experimental and computational investigations into the mechanism of formation of acyl(chloro)phosphines by our synthetic method reveal a pathway in which chloride attacks a phosphonium intermediate and leads to the reductive loss of anthracene from the phosphorus center in a P(v) to P(iii) process. The synthetic applicability of the acyl(chloro)phosphine functional group has been demonstrated by reduction to an acylphosphide anion, which can in turn be treated with an acyl chloride to furnish dissymmetric bis(acyl)phosphines.

## Abstract 2

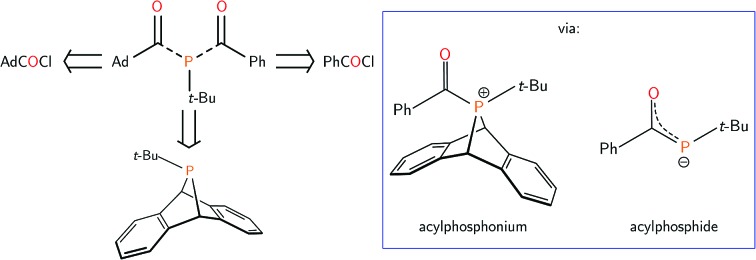
Acyl(chloro)phosphines RC(O)P(Cl)(*t*-Bu) have been prepared by formal insertion of *tert*-butyl phosphinidene (*t*-Bu–P) from *t*-BuP**A** (**A** = C_14_H_10_ or anthracene) into the C–Cl bond of acyl chlorides.

Acylphosphines and their oxides are used as photoinitiators for radical-initiated polymerization^1^–^4^ reactions used in the manufacture of consumer goods such as automotive coatings, adhesives, latex composition kits, and various dental and orthodontic materials.^5^–^14^ Known methodologies for the synthesis of acylphosphines employ nucleophilic sources of phosphorus such as tris(trimethylsilyl)phosphine (P(TMS)_3_, TMS = trimethylsilyl),^15^–^17^ which is volatile, pyrophoric, toxic, expensive, and difficult to handle.^18^ Transition metal-supported phosphines and phosphides have been used as a platform in niche syntheses of acylphosphines, though scope is limited and products remain bound to the metal center.^19^–^23^ Perhaps the most versatile synthetic approach has emerged from the groups of Liotta, Becker, Grützmacher and Mézailles who employ PH_3_ and MPH_2_ (M = Li, Na, K) as nucleophilic sources of phosphorus en route to a wide variety of mono-, bis-, and tris(acyl)phosphines.^24^–^31^ More recently, salts of the 2-phosphaethynolate anion ([PCO]^–^)have been used in the synthesis of functional groups related to acylphosphines.^32^ Despite these advances, enlarging the scope of methods for accessing valuable acylphosphines with different substitution patterns remains an attractive goal.^33^

We have identified dibenzo-7λ^3^-phosphanorbornadienes (RP**A**, **A** = anthracene or C_14_H_10_) as efficient compounds to generate and transfer metastable singlet phosphinidene species.^34^,^35^ Herein, the *tert*-butyl phosphinidene group of *t*-BuP**A** is shown to formally insert into the carbon–chlorine bond of acyl chlorides to generate the rare acyl(chloro)phosphine functional group.^36^,^37^ This product is subsequently exploited for further functionalization.

Treatment of *t*-BuP**A** with benzoyl chloride (PhC(O)Cl) in dichloromethane at 23 °C (Scheme 1) led to an instant colour change from colourless to bright yellow, and the quantitative formation of **1** as a yellow powder, isolated in 82% yield. The species exhibited a singlet resonance at *δ* 114 ppm in the ^31^P{^1^H} NMR spectrum. The ^13^C{^1^H} NMR spectrum of **1** shows a diagnostic doublet resonance in the carbonyl region at *δ* 210 ppm (*J*_PC_ = 67 Hz) while the IR spectrum of **1** displays a complementary C=O stretching band at 1648 cm^–1^. Collectively, these data support the formulation of **1** as (*tert*-butylchlorophosphanyl)(phenyl)methanone, PCl(*t*-Bu)(C(O)Ph).

**Scheme 1:**
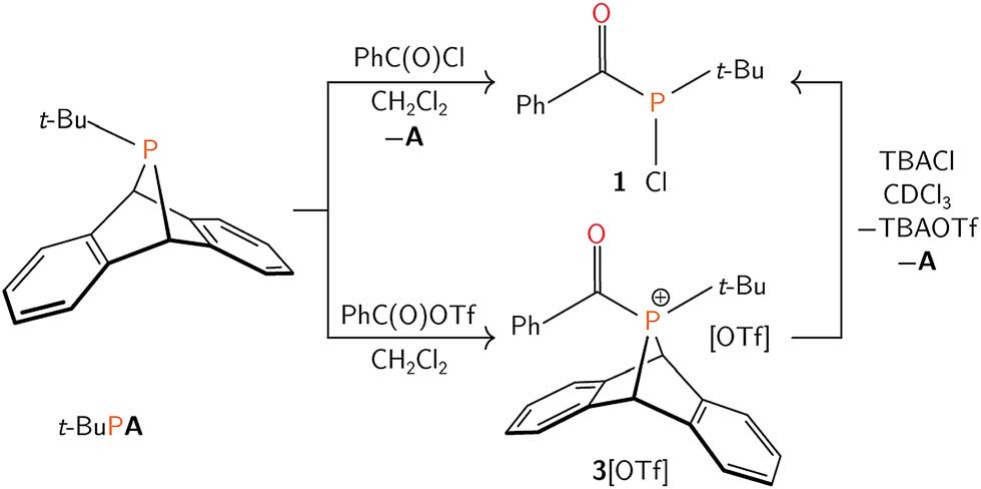
Synthesis of **1** and **3**[OTf].

As **1** could not be isolated as single crystals suitable for structural analysis, a transition metal complex of this phosphine was prepared. Treatment of **1** with half an equivalent of [Ru(*p*-cymene)Cl_2_]_2_ in tetrahydrofuran led to the formation of coordination complex **2**. Layering the reaction mixture with pentane at 23 °C gave **2** as a red-orange crystalline material in 77% yield. Multinuclear NMR spectroscopy studies, in addition to a single crystal X-ray diffraction study confirmed the formulation of **2** as Ru(*p*-cymene)Cl_2_(PCl(*t*-Bu)(C(O)Ph)) (Fig. 1). Racemic **2** crystallizes in the *P*2_1_/*c* space group, and the solid state structure showed C–O, P–C, and P–Ru bond lengths of 1.221(4), 1.875(4) and 2.3178(8) Å respectively, which are in line with the expected values.^38^ Ru–P binding of acylphosphines to ruthenium has been observed in the coordination chemistry of related P(iii) ligands.^39^,^40^

**Fig. 1:**
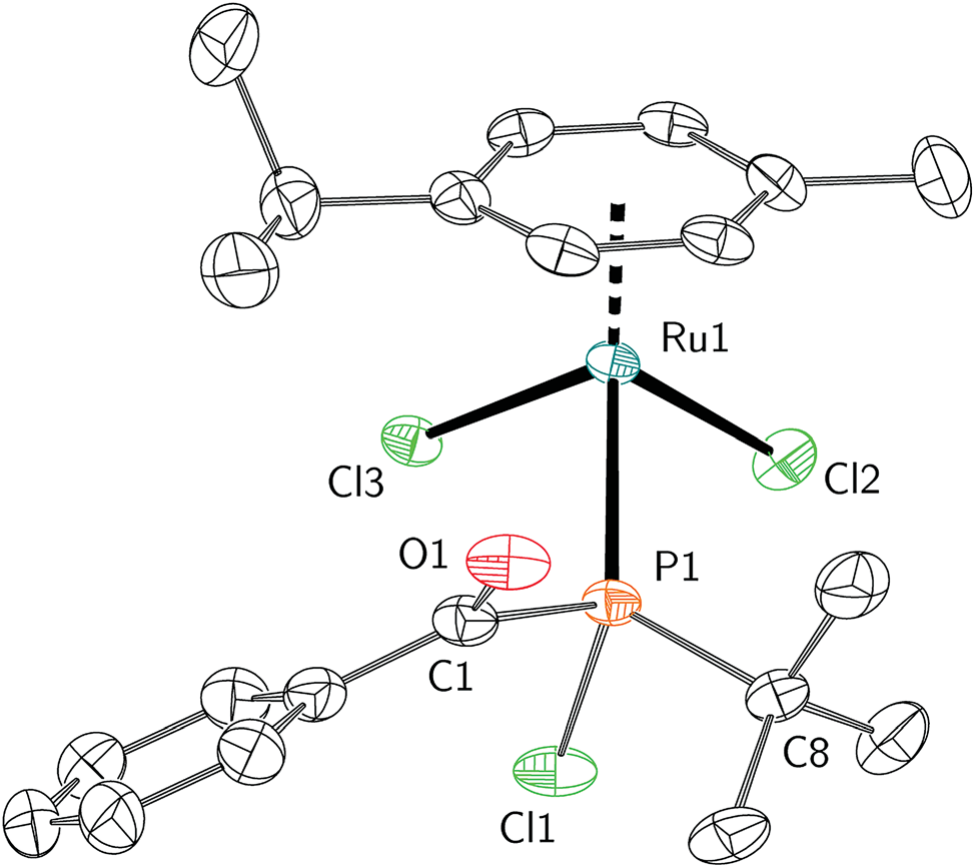
Molecular structure of **2** with ellipsoids set at the 50% probability level and hydrogen atoms omitted for clarity.

Previous studies on thermal phosphinidene transfer have established that thermal loss of a phosphinidene does not occur when the R-group of an RP**A** compound is not π-donating.^35^ Therefore, the mechanism of acylphosphine formation is more likely to proceed associatively *via* nucleophilic attack by phosphorus at the electrophilic carbonyl group of benzoyl chloride. We envisioned that replacement of the chloride for a less nucleophilic anion may lead to the isolation of the putative cationic acylphosphonium intermediate. To this end, the reaction of equimolar solutions of *t*-BuP**A** and benzoyl triflate (PhC(O)OTf, OTf = CF_3_SO_3_^–^)^41^ led to the formation of **3**[OTf] (Scheme 1) as a white powder in 75% yield. A singlet resonance is observed at *δ* 127 ppm in the ^31^P{^1^H} NMR spectrum of **3**[OTf]. The ^13^C{^1^H} NMR spectrum of **3**[OTf] showed a diagnostic carbonyl resonance at 208 ppm and the IR spectrum displayed a C=O absorption peak at 1636 cm^–1^. These data are consistent with the formulation of **3**[OTf] as [**A**P(*t*-Bu)(C(O)Ph)][OTf].

Compound **3**[OTf] decomposes slowly in solution at ambient temperature, yielding an anthracenyl(acyl)hydridophosphonium triflate salt as the major product (see ESI†). Similar C–H activation of arenes by phosphorus-containing electrophiles has been described for metal-stabilized phosphenium triflates.^42^–^44^ Despite its instability in solution, **3**[OTf] can be stored in the solid state at –35 °C for weeks without decomposition and purified by recrystallization from saturated dichloromethane/pentane solutions, also at –35 °C. Treatment of **3**[OTf] with a chloride source (tetra-*n*-butylammonium chloride) in chloroform at 23 °C quantitatively generated **1**, [TBA][OTf] and anthracene (Scheme 1). This reactivity is consistent with nucleophilic attack of the phosphonium center in **3**[OTf] by chloride, inducing reductive loss of anthracene.

The mechanism leading to the formation of **1** was probed *via* competition experiments involving the addition of two different *para*-substituted benzoyl chloride substrates in a 1 : 1 ratio to *t*-BuP**A**. The product ratio was determined by quantitative ^31^P{^1^H} NMR spectroscopy and a series of such experiments is summarised in the Hammett plot (Fig. 2). A small negative value for *ρ* of –2.36 is consistent with build-up of positive charge in the transition state.^45^ This is particularly evident for electron withdrawing groups, such as CF_3_, where the reaction is accelerated compared to electron rich *para*-substituted benzoyl chlorides. These electronic effects are further evidence of phosphine attack at the electrophilic carbon atom of the acylphosphine functional group in the rate limiting step of the reaction.

**Fig. 2:**
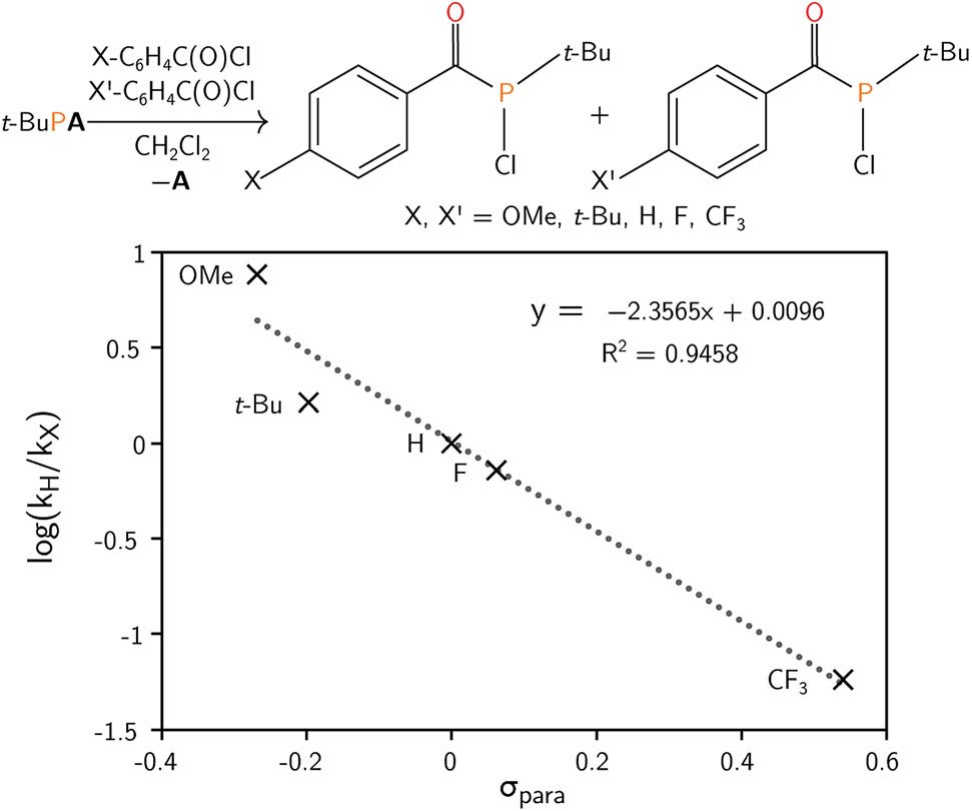
(Top) Preparation of acyl(chloro)phosphines starting from *t*-BuP**A** and *para*-substituted benzoyl chlorides. (Bottom) Hammett plot for the reaction, obtained by competition experiments.

Quantum chemical calculations were employed to study the formation of **1**. These were performed using ORCA 4.0.0.2 46 at the B3LYP/Def2-TZVP level of theory and the results are summarised in Fig. 3. According to our calculations, initial nucleophilic attack of *t*-BuP**A** on benzoyl chloride proceeds with an activation barrier of +17.9 kcal mol^–1^**TS1**, in which the phosphorus atom occupies a tetrahedral environment, leads to an intermediate comprising a phosphonium and chloride ion-pair, **I1**. The distance between the phosphorus and chlorine atoms is 2.55 Å which is significantly longer than the sum of the single-bond covalent radii, 2.10 Å.^38^ However, the phosphorus center features a trigonal bipyramidal structure instead of the ideal tetragonal coordination sphere of a 4-coordinate phosphonium species, suggestive of a strong interaction between the phosphonium centre and chloride. A second transition state in the reaction pathway **TS2** was located, which corresponds to formation of a P–Cl bond and concomitant loss of anthracene to give **1**. **TS2** had a relative Gibbs free energy of +15.6 kcal mol^–1^, which is lower than the Gibbs free energy for **TS1**. The loss of anthracene in **TS2** is highly concerted and appears to proceed by a cheletropic extrusion pathway. This type of formal reductive elimination from λ-5 phosphoranes to give a phosphorus(iii) product has previously been described in the literature.^47^–^50^ Finally, we investigated the possibility of a phosphenium chloride salt intermediate (**I2**, see ESI†), which would arise from loss of anthracene from cation **3** and is the formal reverse of the McCormack reaction.^49^–^51^ We found the relative Gibbs free energy of intermediate **I2** to be 65.0 kcal mol^–1^ which is significantly higher than either **TS1** of **TS2**, ostensibly ruling it out as an intermediate in the reaction mechanism (see ESI†).

**Fig. 3:**
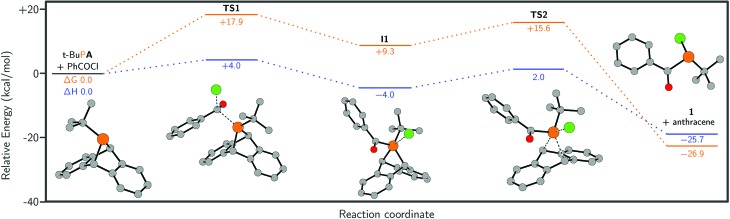
Calculated lowest energy pathway for the reaction of *t*-BuP**A** with benzoyl chloride, at the B3LYP/Def2-TZVP level of theory (see ESI† for full computational details). Δ*G* is shown in orange and Δ*H* is shown in blue. Hydrogen atoms are omitted for clarity.

The higher Gibbs free energy of the first transition state compared to the second is consistent with experiment, where a phosphonium intermediate is not observed when benzoyl chloride is used as the acylating reagent. Thus it is indicated that, when switching from chloride to triflate, there is a change in the rate determining step from **TS1** to **TS2**, which are in proximity (17.9 and 15.6 kcal mol^–1^, respectively) as determined by DFT calculations.

Efforts to further derivatise **1** were undertaken by reduction of the P–Cl bond. Treatment of compound **1** with 2 equivalents of freshly-prepared sodium naphthalenide^52^ led to the formation of a new species with concomitant formation of naphthalene and sodium chloride (Scheme 2). Addition of 15-crown-5 to the reaction mixture led to the isolation of [Na(15-crown-5)]**4** as a crystalline orange powder in 49% yield. The connectivity of anion **4** as [OC(Ph)P*t*-Bu]^–^ was confirmed by a single crystal X-ray diffraction experiment (Fig. 4). In the solid state, the P–C and C–O bond lengths are 1.756(2) and 1.271(2) Å, respectively. The short C–P bond and long C–O bond are consistent with delocalization of electron density from a phosphide lone pair into the π*(C=O) orbital.

**Scheme 2:**
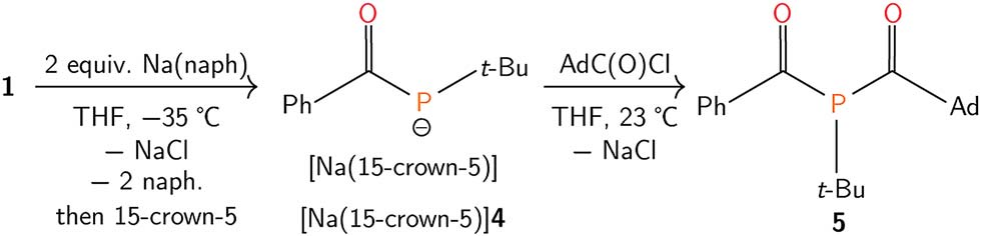
Synthesis of [Na(15-crown-5)]**4** and **5**.

**Fig. 4:**
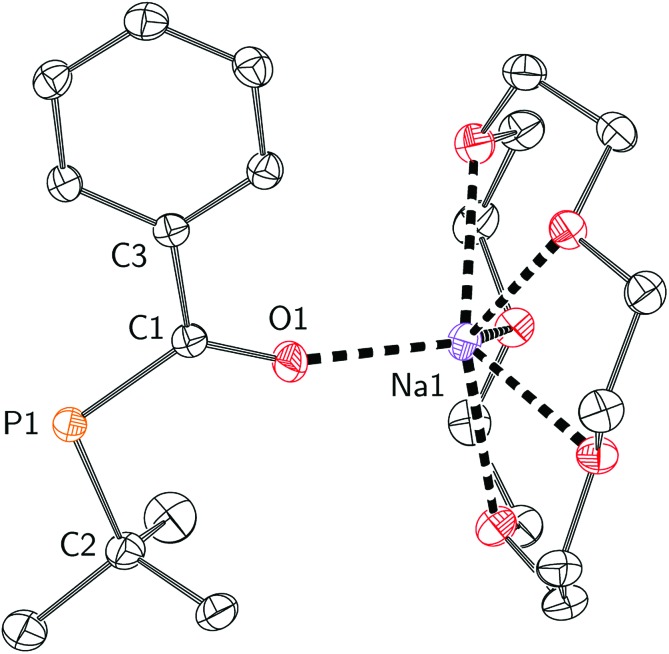
Molecular structure of [Na(15-crown-5)]**4** with ellipsoids set at the 50% probability level and hydrogen atoms omitted for clarity.

To quantify the degree of electron delocalization over the acylphosphide functional group, the electronic structure was evaluated using natural bond orbital (NBO) methods.^53^ Natural resonance theory (NRT) analysis, implemented within the NBO package, showed that the primary resonance structure (**4a**, Fig. 5) had a contribution of 39%. A second resonance structure, containing a carbon-phosphorus double bond and carbon-oxygen single bond (**4b**, Fig. 5), closely followed with a contribution of 33%, while the remaining resonance structures each had less than 2% weight. The C–O and C–P natural bond orders were remarkably similar at 1.51 and 1.49, respectively. The high degree of delocalization of the phosphide lone pair into the carbonyl unit seems to be a result of the anionic charge and of having two lone pairs at P, one of which is higher in energy and has more p-character and is thus more prone to delocalization, since for neutral acylphosphine-containing molecules, minimal delocalization has been observed.^54^,^55^

**Fig. 5:**
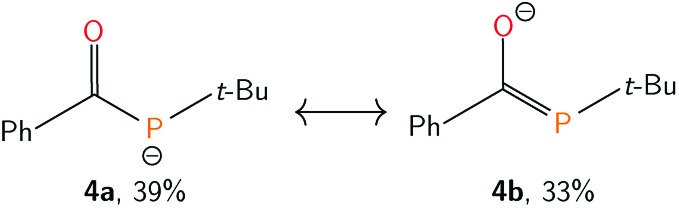
Leading resonance structures of anion **4**, as determined by Natural Resonance Theory (NRT), implemented within the Natural Bond Orbital (NBO) package.

The combination of [Na(15-crown-5)]**4** and AdC(O)Cl (Ad = 1-adamantyl) in tetrahydrofuran generated **5**, which was isolated as a crystalline yellow solid in 55% yield (Scheme 2). In the ^31^P{^1^H} NMR spectrum of **5**, a singlet resonance is observed at *δ* 37 ppm. Two diagnostic doublet resonances are observed in the downfield region of the ^13^C{^1^H} NMR spectrum at *δ* 225 (*J*_PC_ = 50 Hz) and 214 ppm (*J*_PC_ = 46 Hz), which are assigned to the two acyl carbon atoms. The IR spectrum of **5** shows two C=O absorption peaks at 1665 and 1629 cm^–1^. These spectroscopic data inform our formulation of **5** as OC(Ph)P(C(O)Ad)*t*-Bu. Single crystals of **5** were grown from a concentrated pentane solution and analyzed by an X-ray diffraction study, which featured **5** as a single (*R*)-enantiomer (see ESI†) in the chiral space group *P*2_1_ (Fig. 6).

**Fig. 6:**
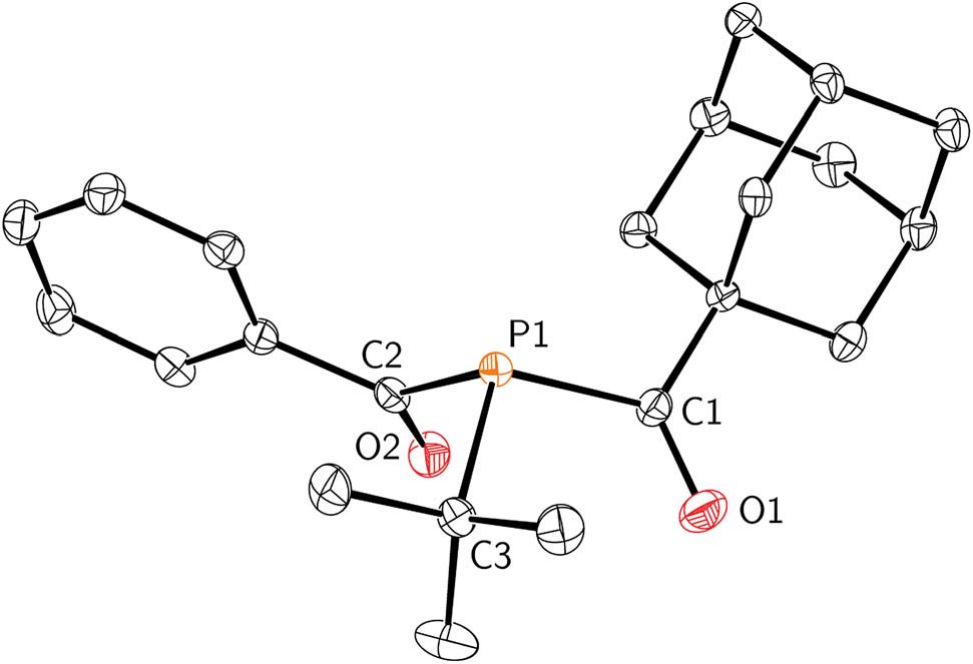
Molecular structure of (*R*)-**5** with ellipsoids set at the 50% probability level and hydrogen atoms omitted for clarity. P1–C1, C1–O1, P1–C2 and C2–O2 bond lengths are 1.209(4), 1.902(3), 1.212(4) and 1.888(3) Å, respectively.

The formation of **5** from [Na(15-crown-5)]**4** provides an approach for preparing dissymmetric bis(acyl)phosphines.^56^ Interestingly, a previous report of a (W(CO)_5_) P-protected acylphosphide anion describes C–O instead of C–P bond formation upon treatment with an acyl chloride, to give an ester-containing phosphaalkene.^57^

In summary, we have described a novel reaction of *t*-BuP**A** with acyl chlorides to give acyl(chloro)phosphine-containing products, which were not accessible previously.^36^,^37^ Experimental mechanistic studies agree well with computational insights, and clearly demonstrate the presence of an intermediate acylphosphonium species. The acyl(chloro)phosphine functional group can be reduced to the corresponding acylphosphide anion, which reacts further with an acyl chloride to provide access to a dissymmetric bis(acyl)phosphine. Other synthetic pathways to related acylphosphine derivatives are the subject of on-going investigations.

## Conflicts of interest

There are no conflicts to declare.

## Supplementary Material

Supplementary information

Crystal structure data
