# Supplementary material for: Synthesis of acyl(chloro)phosphines enabled by phosphinidene transfer
Source: Chem Sci. 2019 Feb 7;10(12):3627–31. doi: 10.1039/c8sc05657a (PMC6432624; doi:10.1039/c8sc05657a)
Supplement: Supplementary file 1 [file SC-010-C8SC05657A-s001.pdf]

Electronic Supplementary Material (ESI) for Chemical Science.
This journal is © The Royal Society of Chemistry 2019

Supporting Information: Synthesis of Acylchlorophosphines Enabled by Phosphinidene Transfer

Kevin M. Szkop,[†,‡] Michael B. Geeson,[†] Douglas W. Stephan,[*,‡] and Christopher C. Cummins[*,†]

†*Department of Chemistry, Massachusetts Institute of Technology, Cambridge, MA 02139, USA*

‡*Department of Chemistry, University of Toronto, 80 St George St, Toronto, Ontario, M5S3H6, Canada*

E-mail: dstephan@chem.utoronto.ca; ccummins@mit.edu

Contents

S1 Synthetic details and characterization of compounds **S-3**

S1.1 General methods S-3

S1.2 Synthesis of **1** S-4

S1.3 Synthesis of **2** S-5

S1.4 Synthesis of **3**[OTf] S-12

S1.5 Formation of **1** from **3**[OTf] S-15

S1.6 Rearrangement of **3**[OTf] S-15

S1.7 Synthesis of [Na(15-crown-5)]**4** S-18

S1.8 Synthesis of **5** S-20

S1.9 Competition experiments S-24

S2 X-ray crystallographic studies **S-29**

S2.1 General methods S-29

S2.2 Compound **2** S-29

S2.3 Compound [Na(15-crown-5)]**4** S-31

S2.4 Compound **5** S-33

S3 Computational studies **S-35**

S3.1 General procedures S-35

S3.2 Natural Bond Order (NBO) analysis S-37

S3.3 Tables of optimized coordinates S-37

S1 Synthetic details and characterization of compounds

S1.1 General methods

All manipulations were performed in a Vacuum Atmospheres model MO-40M glovebox under an inert atmosphere of purified N_2 or using standard Schlenk techniques. When reagents were removed from a stock bottle containing a Sure/Seal, the equivalent volume of dry nitrogen was injected into the bottle prior to removing the desired volume of solution with a syringe. All solvents were obtained anhydrous and oxygen-free by bubble degassing (argon) and purification by passing through columns of alumina and Q5.[S1] Once collected, solvents were stored over activated 4 Å molecular sieves (20 wt%) inside the glovebox.[S2] All glassware was oven-dried for at least 6 h prior to use, at temperatures greater than 150 °C.

Mg**A**-3THF (**A** = anthracene, $C_{14}H_{10}$),[S3] *t*-BuP**A**,[S4] benzoyl triflate[S5] (PhC(O)OTf) and sodium naphthalenide solutions[S6] were prepared according to literature procedures. Benzoyl chloride (Sigma-Aldrich), *para*-substituted benzoyl chloride reagents (Oakwood) and 15-crown-5 (Lancaster) were degassed three times by the freeze-pump-thaw method before bringing into the glovebox and stored over 4 Å molecular sieves prior to use. Dichloro(*p*-cymene)ruthenium(II) dimer ($[Ru(p-cymene)_2Cl_2]_2$, Strem) and 1-adamantanecarbonyl chloride (Sigma-Aldrich) were used as received. Tetra-*n*-butylammonium chloride (TBACl) was recrystallized from THF under an inert atmosphere prior to use. Benzene-d_6 (C_6D_6), acetonitrile-d_3 (CD_3CN) and chloroform-d ($CDCl_3$) were purchased from Cambridge Isotope Labs and were degassed three times by the freeze-pump-thaw method and stored over activated 4 Å molecular sieves for 48 h in the glovebox prior to use. Diatomaceous earth (Celite 435, EM Science), 4 Å molecular sieves (Millipore-

Sigma) and Activated Charcoal Norit CA1 (Aldrich) were dried by heating to 200 °C under dynamic vacuum for at least 48 h prior to use. The temperature of the aluminum shot used to heat reagents or reaction mixtures was measured using a Hanna Instruments K-type Thermocouple Thermometer (model HI935005).

NMR spectra were obtained on Varian Inova 300 and 500 instruments equipped with Oxford Instruments superconducting magnets, on a Jeol ECZ-500 instrument equipped with an Oxford Instruments superconducting magnet, or on a Bruker Avance 400 instrument equipped with a Magnex Scientific or with a SpectroSpin superconducting magnet. ^{1}H and ^{13}C NMR spectra were referenced to residual benzene-d_6 (^{1}H = 7.16 ppm, ^{13}C = 128.06 ppm), acetonitrile-d_3 (^{1}H = 1.94 ppm, ^{13}C = 118.26 ppm) or chloroform-d (^{1}H = 7.26 ppm, ^{13}C = 77.16 ppm). ^{19}F NMR spectra were referenced externally to $CFCl_3$ (0 ppm). ^{31}P NMR spectra were referenced externally to 85% H_3PO_4 (0 ppm).

Infrared spectra were collected using a Bruker ATR-IR Tensor 37. Samples were removed from the glovebox in sealed vials and briefly handled in air prior to data collection.

High resolution mass spectral (HRMS) data were collected using a Jeol AccuTOF 4G LC-Plus mass spectrometer equipped with an Ion-Sense DART source. Data were calibrated to a sample of PEG-600 and were collected in positive-ion mode. Samples were prepared in DCM (10 μM concentration) and were briefly exposed to air (<5 s) before being placed in front of the DART source.

Elemental combustion analyses were performed by Midwest Micro Laboratories (Indianapolis, IN, USA).

S1.2 Synthesis of 1

A solution of *t*-BuP**A** (0.344 g, 1.29 mmol, 1 equiv.) in CH_2Cl_2 (1 mL) was added to vial containing a Teflon coated stir bar and a solution of benzoyl chloride (PhC(O)Cl,

0.200 g, 1.42 mmol, 1.1 equiv.) in CH_2Cl_2 (1 mL). The solution became bright yellow within seconds, and a white crystalline precipitate (anthracene) appeared within minutes. The reaction mixture was stirred for *ca.* 3 hours at 23 °C. All volatiles were removed *in vacuo*, and the yellow residue was dissolved in diethyl ether (1 mL) and passed through a Celite/charcoal plug. Additional diethyl ether (*ca.* 10 mL) was used to elute the product from the charcoal plug. Volatiles were removed *in vacuo* once more, yielding compound **1** as an analytically pure yellow powder. Isolated yield: 82% yield (0.243 g, 1.0 mmol). ^{1}H NMR (C_6D_6, 400 MHz, 23 °C) δ 7.82 (m, 2H), 7.05 (m, 1H), 6.97 (m, 2H), 1.01 (d, J_{PC} = 13 Hz) ppm. ^{13}C{^{1}H} NMR (C_6D_6, 98 MHz, 23 °C) δ 210.7 (d, J_{PC} = 67 Hz, C=O), 140.2 (d, J_{PC} = 34 Hz, Ar), 133.6 (Ar), 128.8 (Ar), 128.5 (d, J_{PC} = 10 Hz, Ar), 36.2 (d, J_{PC} = 32 Hz, $C(CH_3)_3$), 26.7 (d, J_{PC} = 15 Hz, $C(CH_3)_3$) ppm. ^{31}P{^{1}H} NMR (C_6D_6, 161 MHz, 23 °C) δ 114 ppm. ATR IR: 1648 cm^{-1} (C=O). DART HRMS (positive mode): Compound **1** hydrolyzed under the HRMS experimental conditions. The mass observed corresponds to the replacement of the P-Cl bond for a P-OH bond. Calculated for $C_{11}H_{16}P_1O_2$: 211.088793. Observed: 211.088577. Elemental Analysis: Calculated (found) for $C_{11}H_{14}OPCl$: C, 57.78 (58.01); H, 6.17 (5.98); N, 0.00 (< 0.01).

S1.3 Synthesis of 2

A solution of **1** (25.0 mg, 0.11 mmol, 1 equiv.) in THF (1 mL) was added to vial containing a Teflon coated stir bar and a solution of $[Ru(p\text{–}cymene)_2Cl_2]_2$ (34.0 mg, 0.06 mmol, 0.5 equiv.) in THF (1 mL). The deep red solution was stirred at 23 °C for 1 hour, then all volatiles were removed *in vacuo*. The red residue was washed with pentane (2 × 3 mL), then dried *in vacuo*. The red residue was redissolved in CH_2Cl_2 (1.5 mL) and this solution was layered with pentane (8 mL) at 23 °C for 24 hours. After this time, a crop of red crystals formed in the vial. The supernatant was decanted and the red crystals washed

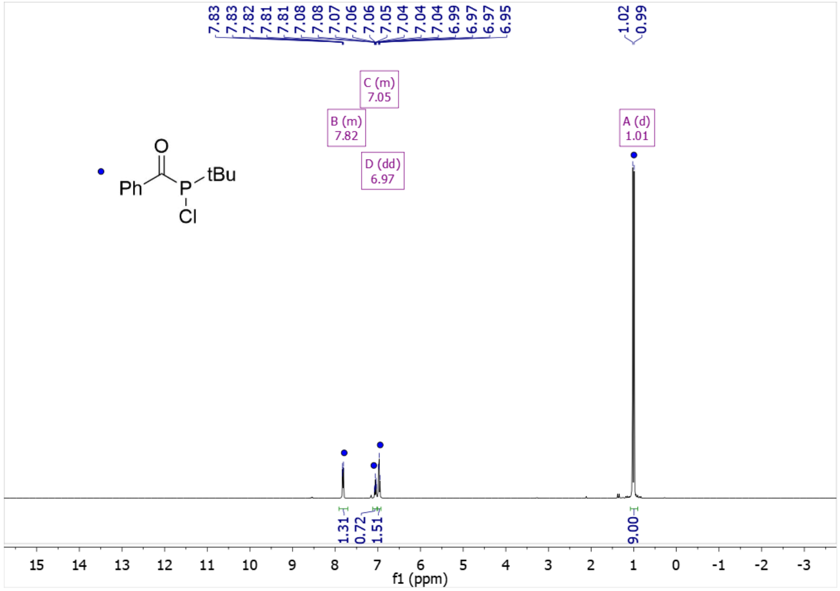

Figure S1: ^{1}H NMR spectrum of **1** in C_6D_6 at 25 °C, recorded at 400 MHz.

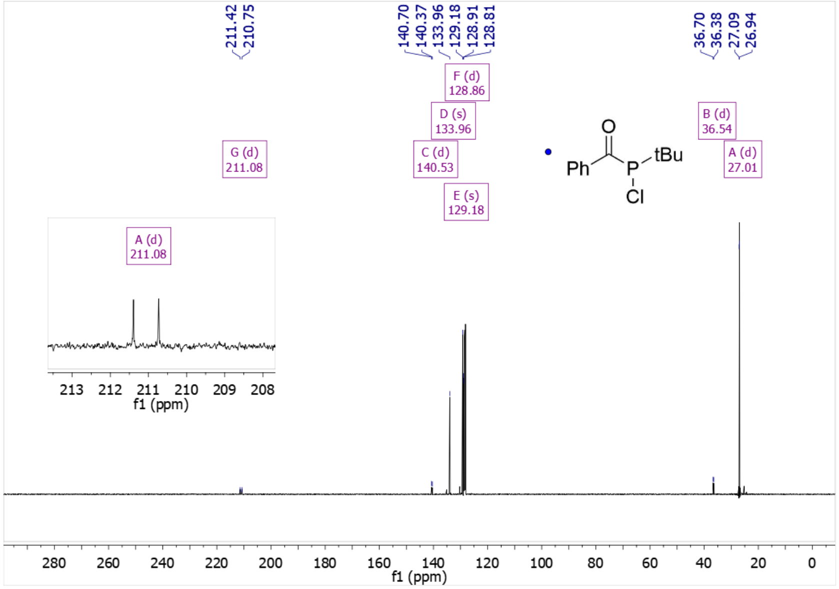

Figure S2: $^{13}C\{^{1}H\}$ NMR spectrum of **1** in C_6D_6 at 25 °C, recorded at 98 MHz. Insert: Downfield region of the $^{13}C\{^{1}H\}$ NMR spectrum of **1**.

with pentane (2 × 3 mL), and dried *in vacuo*. Compound **2** was isolated as a red-orange powder. Isolated yield: 77% yield (46.2 mg, 0.08 mmol). ^{1}H NMR ($CDCl_3$, 400 MHz,

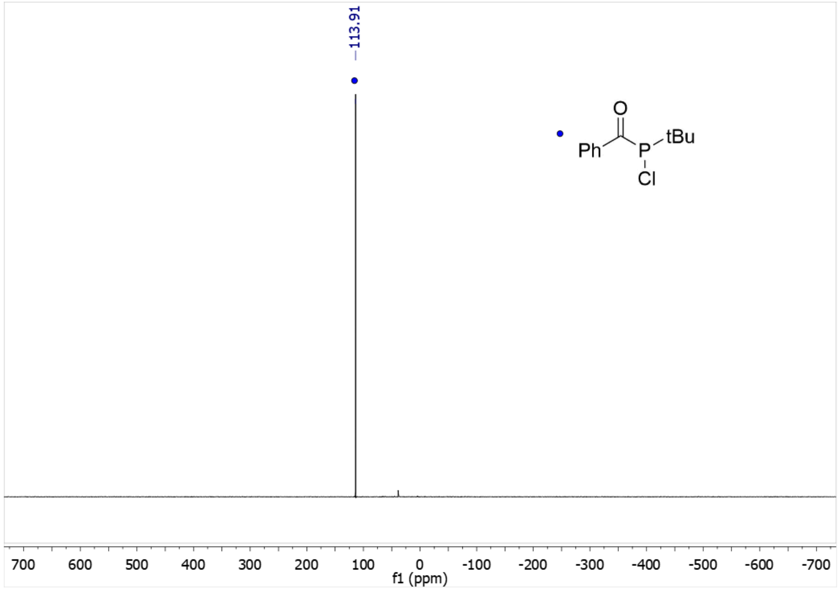

Figure S3: $^{31}P\{^1H\}$ NMR spectrum of **1** in C_6D_6 at 25 °C, recorded at 161 MHz.

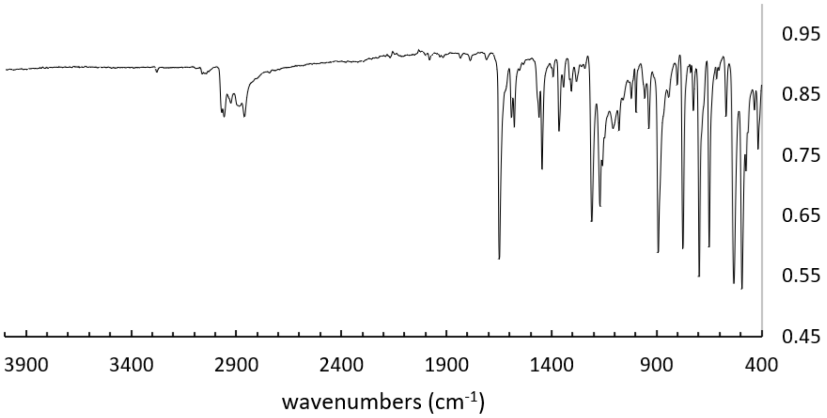

Figure S4: ATR IR spectrum of **1**.

23 °C) δ 8.18 (m, 2H), 7.58 (m, 1H), 7.47 (m, 2H), 5.77 (m, 1H), 5.61 (m, 1H), 5.57 (m, 2H), 2.87 (sept, J_{HH} = 7 Hz), 1.98 (s, 3H, CH_3), 1.45 (d, J_{PH} = 16 Hz, 9H, $C(CH_3)_3$), 1.20 (d, J_{HH} = 7 Hz, 3H, *i*-Pr), 1.18 (d, J_{HH} = 7 Hz, 3H, *i*-Pr) ppm. $^{13}C\{^1H\}$ NMR ($CDCl_3$, 98 MHz, 23 °C) δ 208.3 (s, J_{PC} is not resolved, C=O), 136.9 (d, J_{PC} = 38 Hz, Ar), 133.8 (Ar), 130.3 (Ar), 128.4 (Ar), 92.4 (Ar), 90.7 (d, J_{PC} = 5 Hz, Ar), 87.2 (d,

JPC = 7 Hz, Ar), 86.0 (Ar), 45.8 (s), 30.1 (s), 28.0 (d, J_{PC} = 4 Hz), 22.1 (d, J_{PC} = 98 Hz), 17.4 (s) ppm. ^{31}P{^{1}H} NMR ($CDCl_3$, 161 MHz, 23 °C) δ 135 ppm. ATR IR: 1636 cm^{-1} (C=O). Q-TOF MS (positive mode): Compound **2** fragments into multiple species under MS conditions; the major species corresponds to [**2**·MeCN]$^+$ ([**M**]). The theoretical isotope pattern for this species did not give a perfect match with experiment. However, the experimental spectrum could be fit to a linear combination of 0.2 [**M**] + 0.2 [**M**+1] + 0.2 [**M**+2] + 0.4 [**M**+4]. Despite recrystallization, material of sufficient purity for elemental analysis could not be obtained. Results from the analysis are given for completeness. Elemental Analysis: Calculated (found) for $C_{22}H_{31}OPCl_3Ru$: C, 48.05 (44.23); H, 5.68 (4.73); N, 0.00 (< 0.01).

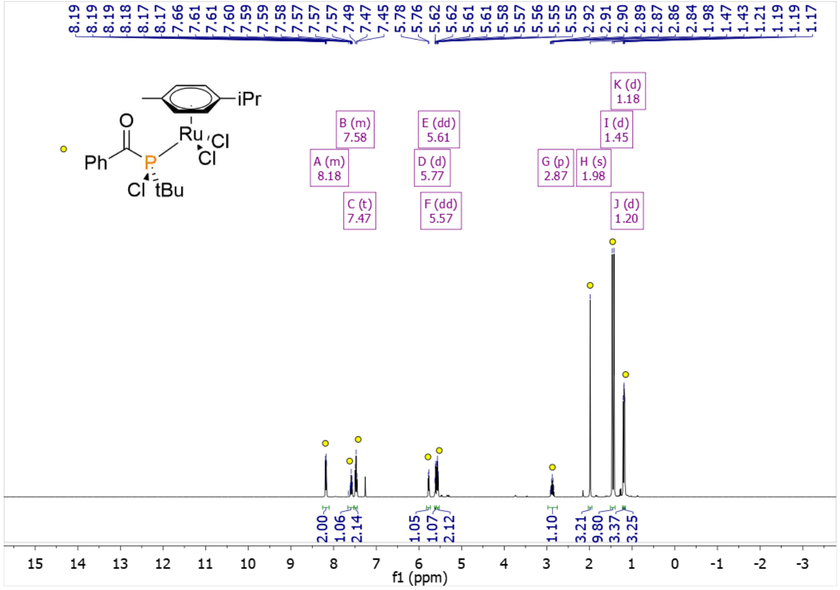

Figure S5: ^{1}H NMR spectrum of **2** in $CDCl_3$ at 25 °C, recorded at 400 MHz.

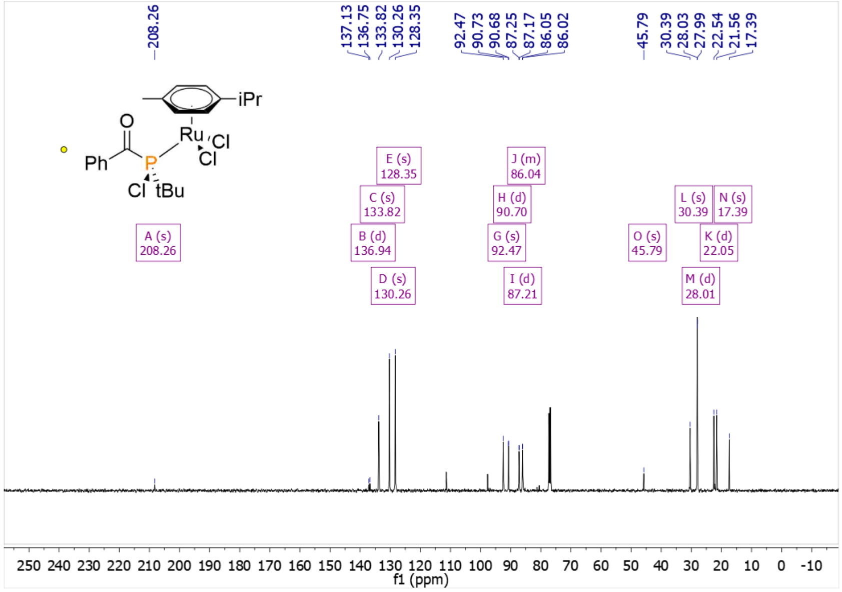

Figure S6: $^{13}C\{^{1}H\}$ NMR spectrum of **2** in $CDCl_3$ at 25 °C, recorded at 98 MHz.

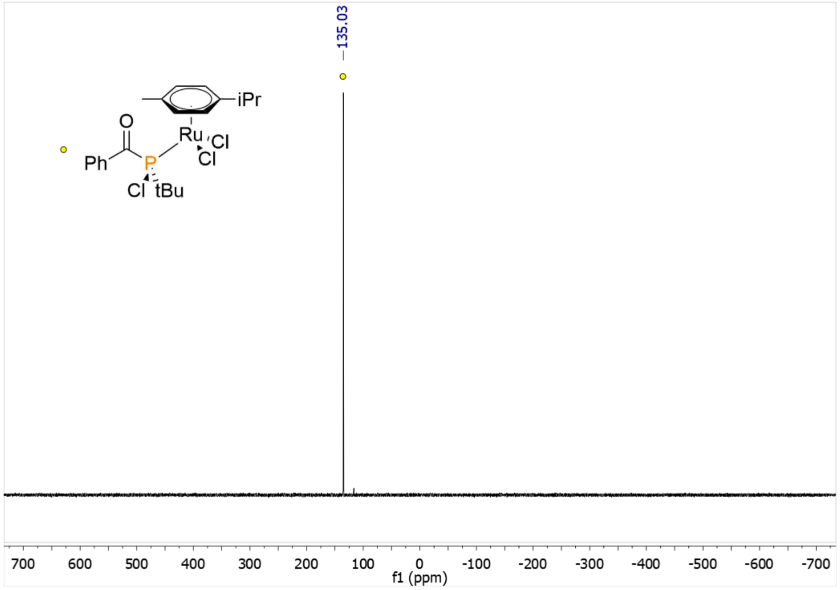

Figure S7: $^{31}P\{^{1}H\}$ NMR spectrum of **2** in $CDCl_3$ at 25 °C, recorded at 161 MHz.

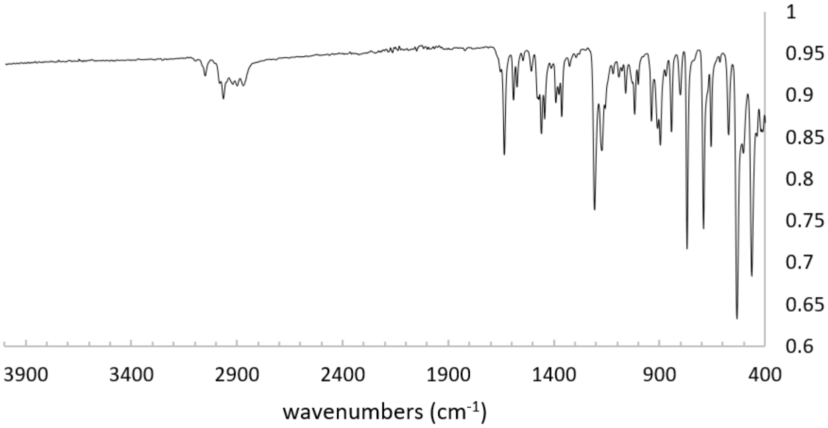

Figure S8: ATR IR spectrum of **2**.

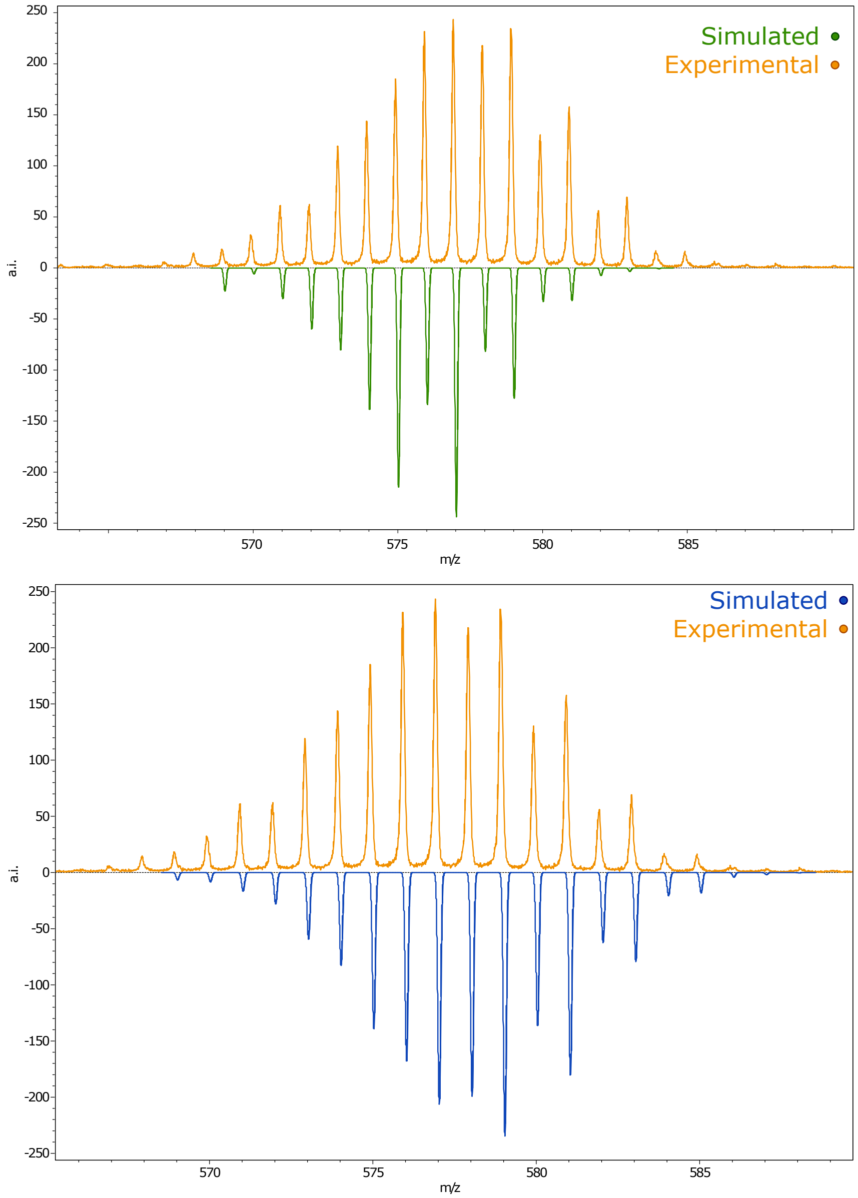

Figure S9: Comparison of the simulated and experimental ESI-MS data for compound **2**. The top fit is to [**M**] (see text for discussion) while the bottom fit is to a linear combination of 0.2 [**M**] + 0.2 [**M**+1] + 0.2 [**M**+2] + 0.4 [**M**+4].

S1.4 Synthesis of **3**[OTf]

A solution of *t*-BuP**A** (22.6 mg, 0.09 mmol, 1 equiv.) in pentane (1 mL) was added to vial containing a Teflon coated stir bar and a solution of benzoyl triflate (PhC(O)OTf, 21.6 mg, 0.09 mmol, 1.0 equiv.) in pentane (1 mL). An insoluble white solid formed immediately. The clear and colourless supernatant was decanted, and the white solid was washed with pentane (1 × 5 mL). The white solid was dried *in vacuo*, then redissolved in CH_2Cl_2 (1.5 mL), forming a clear and colourless solution. Pentane (*ca.* 3 mL) was added and the solution was placed in the freezer at −35 °C for 24 hours. After this time, a crystalline white solid formed. The supernatant was removed and the white solid washed with pentane (3 × 3 mL) then dried *in vacuo*, yielding compound **3**[OTf] as white powder. Isolated yield: 75% yield (33.1 mg, 0.064 mmol). ^{1}H NMR (CD_3CN, 400 MHz, 23 °C) δ 7.86 (m, 3H), 7.76 (m, 2H), 7.67 (m, 2H), 7.44 (m, 4H), 7.10 (m, 2H), 5.89 (d, J_{PH} = 4 Hz, 2H), 1.21 (d, J_{PH} = 17 Hz, 9H) ppm. ^{13}C{^{1}H} NMR (CD_3CN, 98 MHz, 23 °C) δ 193.4 (d, J_{PC} = 20 Hz, C=O), 139.7.2 (d, J_{PC} = 8 Hz, Ar), 138.5 (Ar), 137.8 (Ar), 134.3 (d, J_{PC} = 44 Hz, Ar), 130.1 (Ar), 129.6 (Ar), 129.5 (Ar), 129.0 (Ar), 125.1 (d, J_{PC} = 7 Hz, Ar), 124.8 (d, J_{PC} = 7 Hz, Ar), 49.5 (d, J_{PC} = 34 Hz, $C_{(bridgehead)}$-*H*), 38.5 (d, J_{PC} = 2 Hz, *C*(CH_3)$_3$), 26.1 (C(*C*H_3)$_3$) ppm. ^{19}F{^{1}H} NMR (CD_3CN, 376 MHz, 23 °C) δ -79 ppm. ^{31}P{^{1}H} NMR (CD_3CN, 161 MHz, 23 °C) δ 127 ppm. ATR IR: 1656 cm^{-1} (C=O). Q-TOF MS (positive mode): Calculated for $C_{25}H_{24}OP$: 371.1565. Observed: 370.9215. Elemental Analysis: Calculated (found) for $C_{26}H_{24}F_3O_4PS$: C, 60.00 (59.89); H, 4.65 (4.46); N, 0.00 (< 0.01).

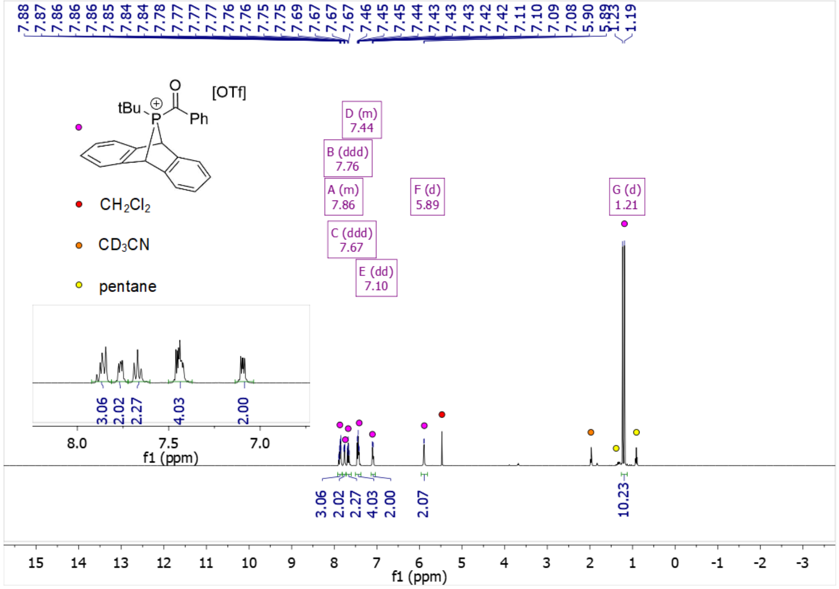

Figure S10: ^{1}H NMR spectrum of **3**[OTf] in CD_3CN at 25 °C, recorded at 400 MHz. Insert: Downfield region of the ^{1}H NMR spectrum of **3**[OTf].

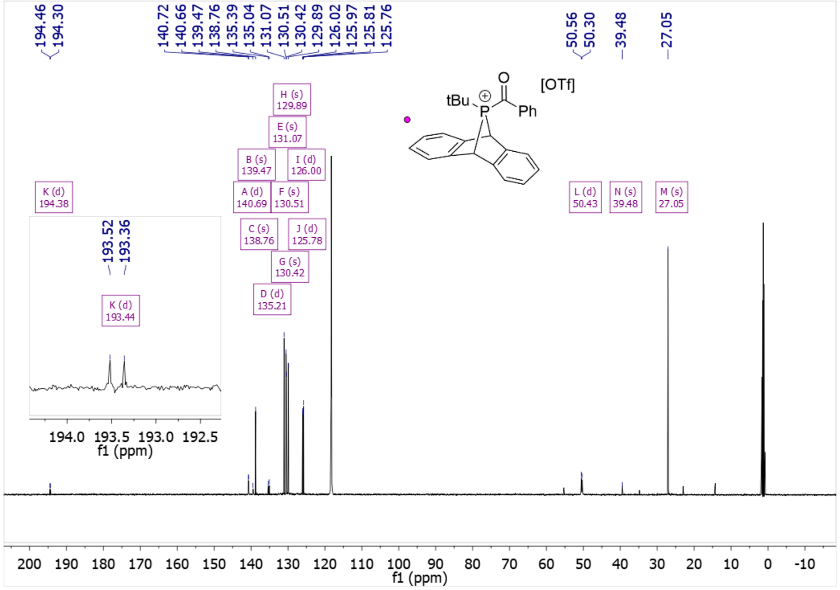

Figure S11: ^{13}C{^{1}H} NMR spectrum of **3**[OTf] in CD_3CN at 25 °C, recorded at 98 MHz. Insert: Downfield region of the ^{13}C{^{1}H} NMR spectrum of **3**[OTf].

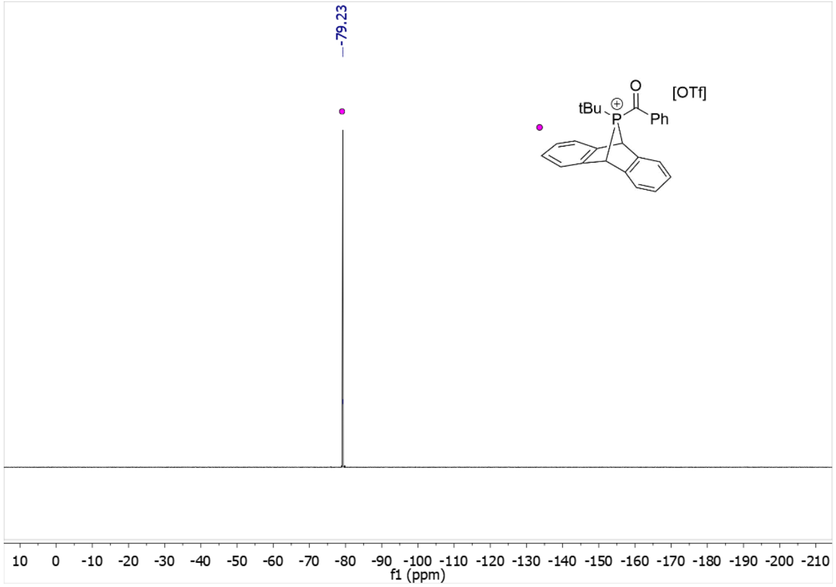

Figure S12: $^{19}F\{^{1}H\}$ NMR spectrum of **3**[OTf] in CD_3CN at 25 °C, recorded at 376 MHz.

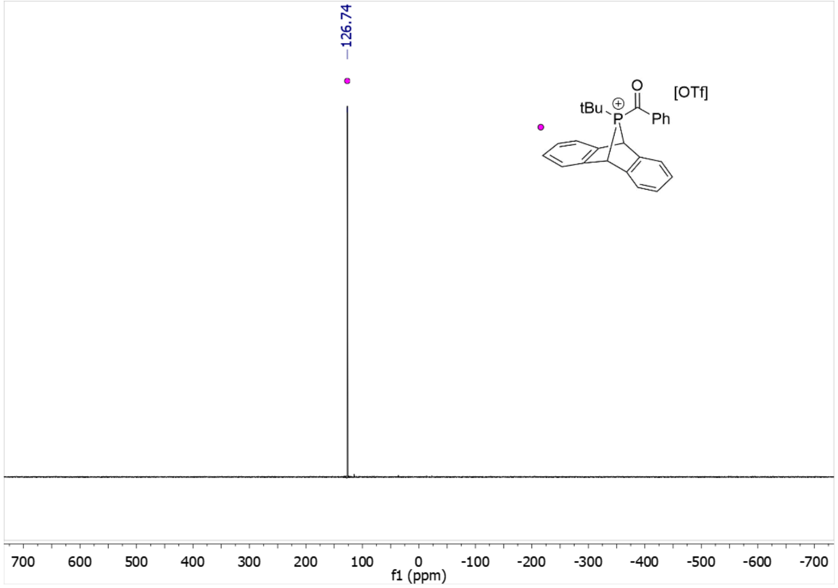

Figure S13: $^{31}P\{^{1}H\}$ NMR spectrum of **3**[OTf] in CD_3CN at 25 °C, recorded at 161 MHz.

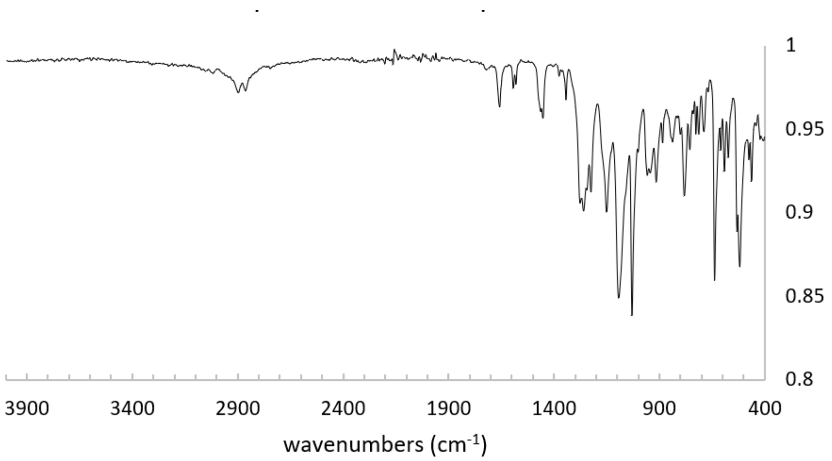

Figure S14: ATR IR spectrum of **3**[OTf].

S1.5 Formation of 1 from 3[OTf]

Compound **3**[OTf] (33.3 mg, 0.064 mmol, 1 equiv.) in $CDCl_3$ (1 mL) was added to a vial containing solid TBACl (tetra-*n*-butylammonium chloride, 20.0 mg, 0.070 mmol, 1.1 equiv.). The reaction mixture immediately turned bright yellow, and was transferred to an NMR tube for analysis. Multinuclear NMR experiments showed the complete consumption of compound **3**[OTf] and the formation of compound **1**, [TBA][OTf] and anthracene.

S1.6 Rearrangement of 3[OTf]

Solutions of freshly-made **3**[OTf] in CH_2Cl_2, CD_3CN or $CDCl_3$ slowly changed from colourless to yellow. Following this reaction by ^{31}P and $^{31}P\{^1H\}$ NMR spectroscopy allowed us to tentatively assign the major products formed. In CH_2Cl_2, consumption of **3**[OTf] is complete after *ca.* 36 hours at 23 °C or *ca.* 90 minutes at 60 °C. Assignments of putative products were made based on chemical shift and coupling constants; specifically, diagnostic $^1J_{PH}$ and $^3J_{PH}$ coupling constants were used to infer the num-

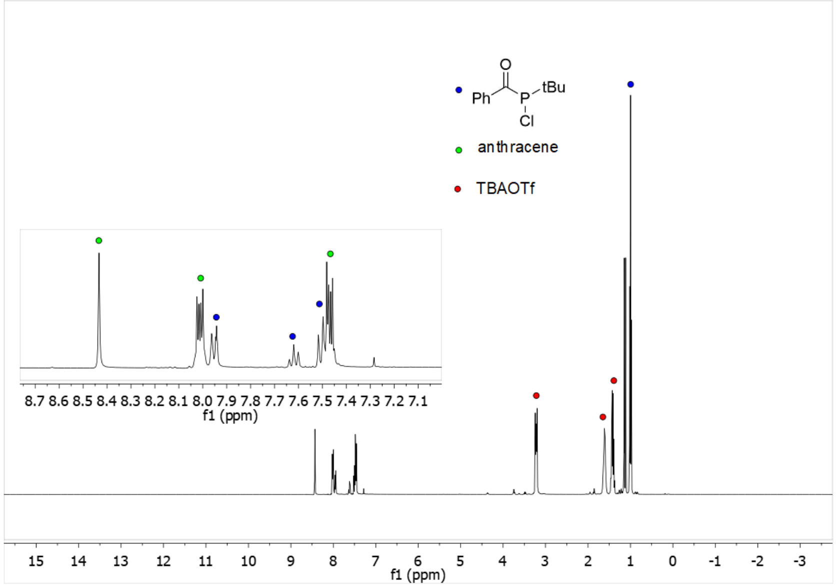

Figure S15: ^{1}H NMR spectrum of reaction mixture containing **1**, anthracene and TBAOTf in $CDCl_3$ at 25 °C, recorded at 400 MHz.

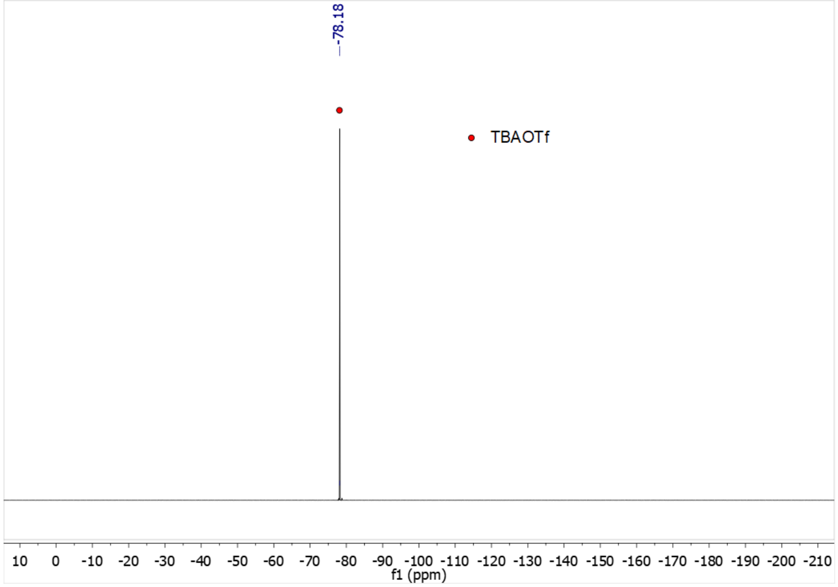

Figure S16: ^{19}F{^{1}H} NMR spectrum of reaction mixture containing **1**, anthracene and TBAOTf in $CDCl_3$ at 25 °C, recorded at 376 MHz.

ber of P-H bonds and the P-*t*-Bu connectivity. We postulate the mechanism (Fig. S18) commences by reductive anthracene loss from **3**[OTf], forming a highly reactive and thus

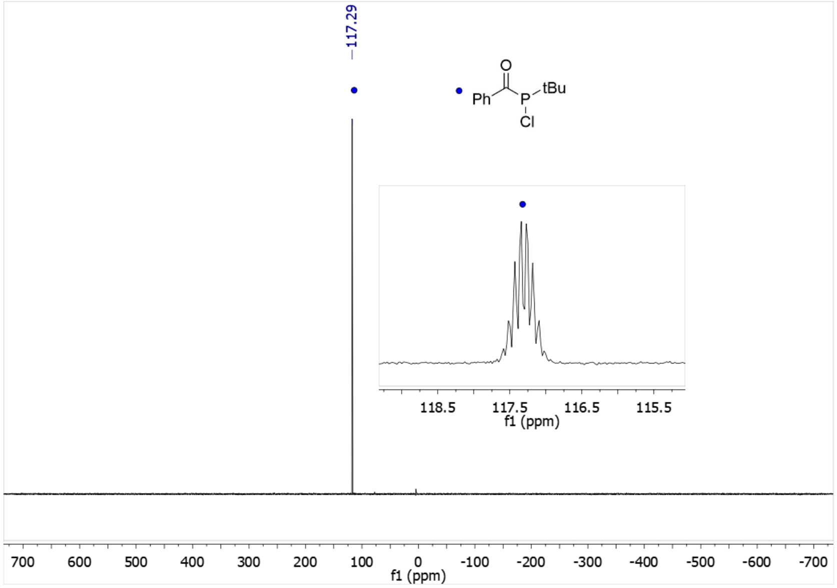

Figure S17: $^{31}P\{^{1}H\}$ NMR spectrum of reaction mixture containing **1**, anthracene and TBAOTf in $CDCl_3$ at 25 °C, recorded at 161 MHz.

unobserved acylphosphenium cation **A**[OTf]. This reactive intermediate then undergoes C-H activation of the released anthacene via a Friedel-Crafts mechanism. Related compounds are known to undergo C-H activation on activated arenes such as naphthalene and biphenyl.[S7–S9] The dearomatized cationic intermediate undergoes a proton migration, which may be triflate-mediated, shifting the proton to the phosphorus centre to yield hydridophosphonium triflate **B**[OTf] as the major product. The addition of excess triethylamine to the reaction mixture is accompanied by a downfield shift and loss of $^{1}J_{PH}$ in the ^{31}P and $^{31}P\{^{1}H\}$ NMR spectra, consistent with deprotonation at phosphorus, to yield phosphine **C**. While unknown in the literature, the ^{31}P NMR chemical shift of putative **C** is consistent with structurally-related acylphosphines.[S10]

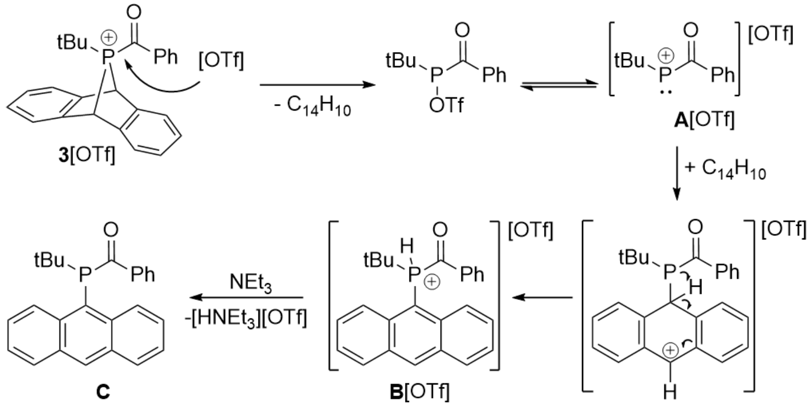

Figure S18: Putative mechanism of rearrangement of **3**[OTf] to **B**[OTf] and subsequent deprotonation to **C**.

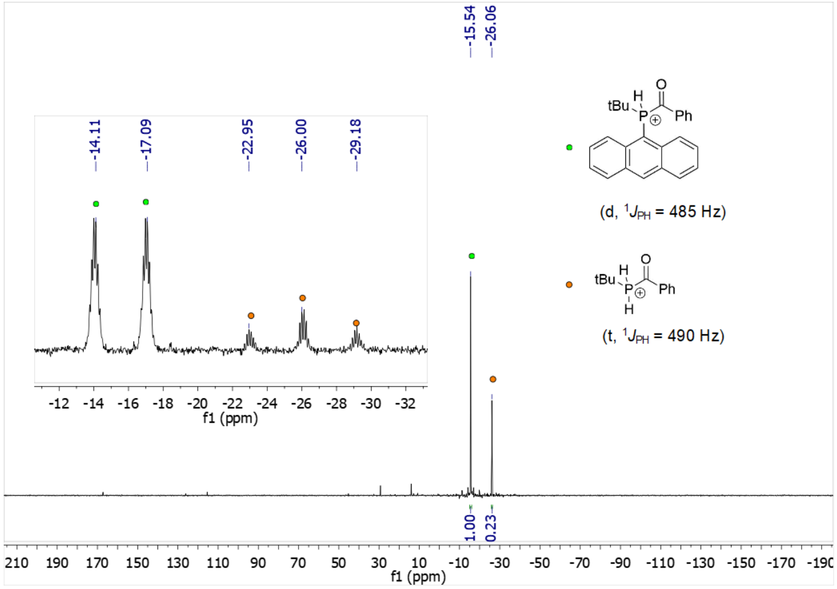

Figure S19: $^{31}P\{^1H\}$ NMR spectrum of a solution of **3**[OTf] after heating to 60 °C for 90 minutes in CH_2Cl_2 at 25 °C, recorded at 161 MHz.

S1.7 Synthesis of [Na(15-crown-5)]4

In a 20 mL scintillation vial charged with a Teflon-coated stir bar, compound **1** (83.3 mg, 0.49 mmol, 1 equiv.) was dissolved in THF (5 mL) and cooled to –35 °C. A deep green

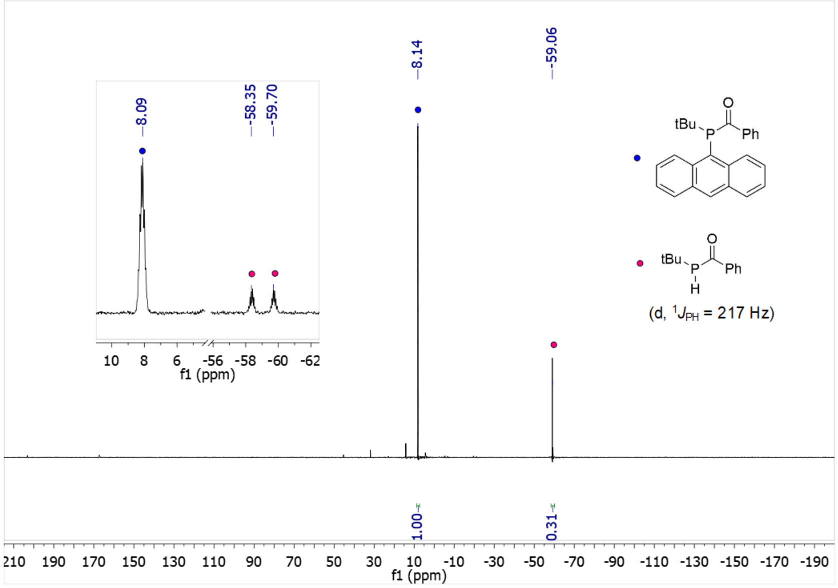

Figure S20: $^{31}P\{^{1}H\}$ NMR spectrum of a solution of **3**[OTf] after heating to 60 °C for 90 minutes with excess NEt_3 in CH_2Cl_2 at 25 °C, recorded at 161 MHz.

solution of freshly made sodium naphthalenide (0.118 M in THF) was cooled to −35 °C. Once both solutions were cooled, the sodium naphthalenide solution was added dropwise to the stirring solution of **1**. After the addition was complete, the reaction mixture was heterogeneous and orange. The solvent was removed *in vacuo* yielding an orange oil, which was extracted with *ca.* 10 mL of a 1:1 diethyl ether/pentane solution, then filtered through a Celite plug. The resulting orange-yellow solution was concentrated by half, then 15-crown-5 (80.2 mg, 0.49 mmol, 1 equiv.) was added, causing an orange solid to precipitate. The supernantant was decanted and the orange solid washed with pentane (2 × 5 mL) and diethyl ether (2 × 5 mL), then dried *in vacuo*. Isolated yield of crude: 70% yield (111.3 mg, 0.26 mmol). This material contains *ca.* 5% of a known diphosphine impurity, $[(t\text{-Bu})(C(O)Ph)P]_2$. For further purification and diffraction-quality single crystals of [Na(15-crown-5)]**4**, CH_3CN solutions of the crude material can be layered with diethyl ether at ambient temperature for 24 hours, yielding a crystalline orange product. Isolated

yield: 49% (78.2 mg, 0.18 mmol). ^{1}H NMR (CD_3CN, 400 MHz, 23 °C) δ 7.80 (m, 2H), 7.13 (m, 3H), 3.63 (s, 20H), 1.29 (d, J_{PH} = 9 Hz, 9H) ppm. ^{13}C{^{1}H} NMR (CD_3CN, 98 MHz, 23 °C) δ 215.5 (d, J_{PC} = 72 Hz, C-O), 149.5 (d, J_{PC} = 46 Hz, Ar), 126.8 (Ar), 126.6 (d, J_{PC} = 4 Hz, Ar), 124.3 (d, J_{PC} = 16 Hz, Ar), 68.6 (s, 15-crown-5), 30.8 (d, J_{PC} = 11 Hz, $C(CH_3)_3$), 29.0 (d, J_{PC} = 31 Hz, $C(CH_3)_3$) ppm. ^{31}P{^{1}H} NMR (CD_3CN, 161 MHz, 23 °C) δ 101 ppm. Q-TOF MS (negative mode): Calculated for $C_{11}H_{14}OP$: 193.0782. Observed: 193.0261. Elemental Analysis: Calculated (found) for $C_{21}H_{34}NaO_6P$: C, 57.79 (57.51); H, 7.85 (7.75); N, 0.00 (< 0.01).

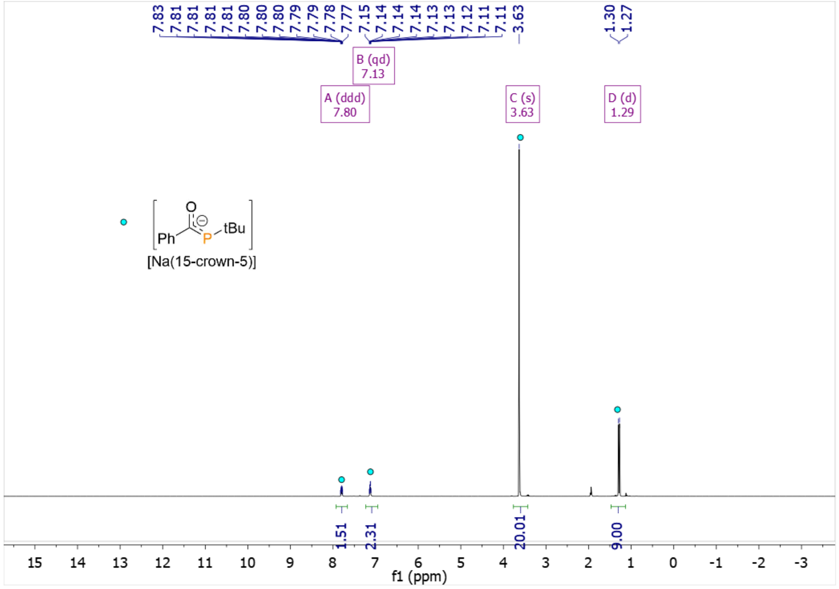

Figure S21: ^{1}H NMR spectrum of [Na(15-crown-5)]**4** in CD_3CN at 25 °C, recorded at 400 MHz.

S1.8 Synthesis of 5

A solution of 1-adamantanecarbonyl chloride (21.0 mg, 0.11 mmol, 1 equiv.) in THF (1 mL) was added to a 20 mL scintillation vial charged with a Teflon-coated stir bar and a solution of [Na(15-crown-5)]**4** (46.1 mg, 0.11 mmol, 1 equiv.) in THF (2 mL). The

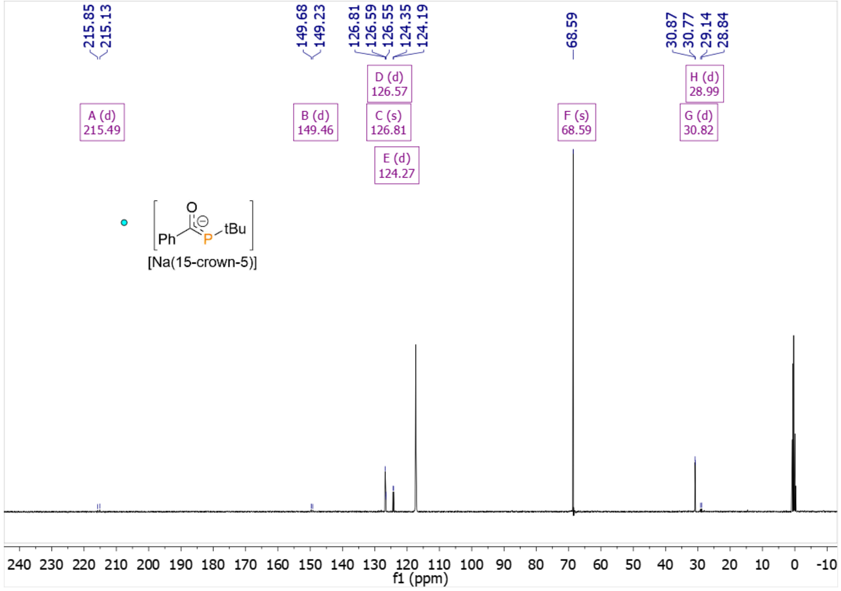

Figure S22: $^{13}C\{^{1}H\}$ NMR spectrum of [Na(15-crown-5)]**4** in CD_3CN at 25 °C, recorded at 98 MHz.

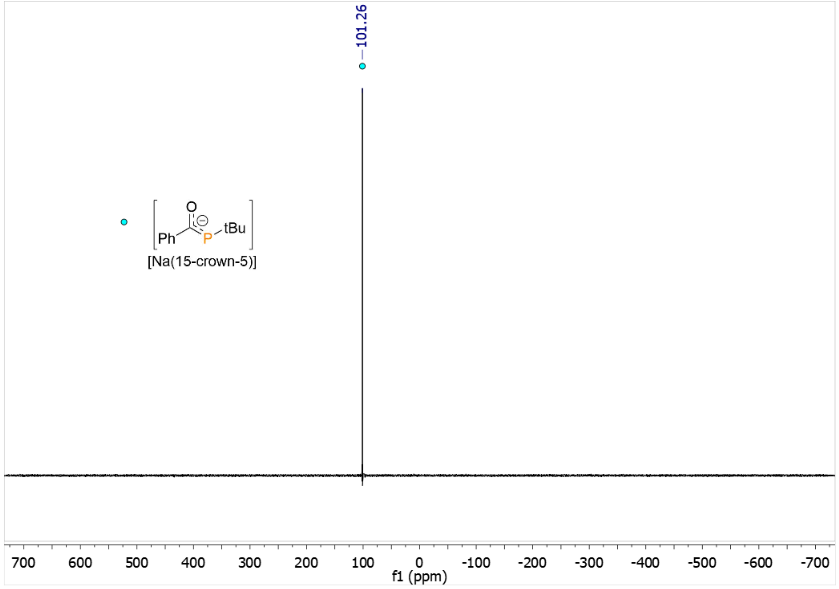

Figure S23: $^{31}P\{^{1}H\}$ NMR spectrum of [Na(15-crown-5)]**4** in CD_3CN at 25 °C, recorded at 161 MHz.

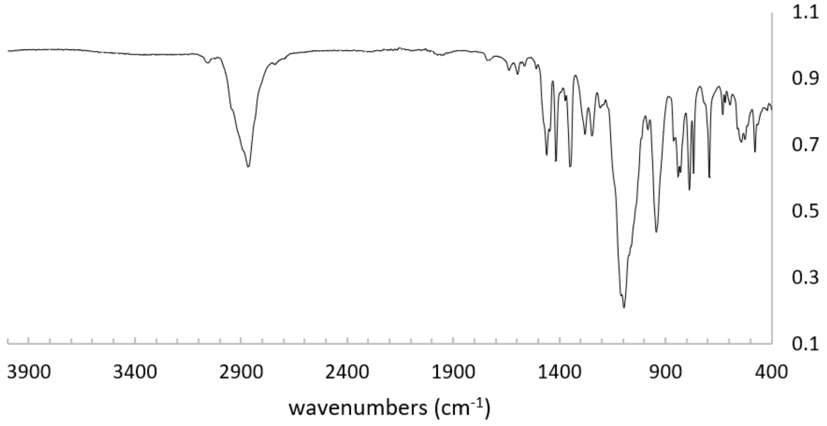

Figure S24: ATR IR spectrum of [Na(15-crown-5)]**4**.

reaction mixture turned pale yellow and a white precipitate formed. After 15 minutes of stirring, volatiles were removed *in vacuo*, yielding a yellow oil. This residue was dissolved in pentane (8 mL), and filtered through a Celite plug. At this point, the mixture consists of **5** and 15-crown-5. The clear yellow solution was concentrated to *ca.* 0.5 mL, then cooled to −35 °C overnight, yielding a crop of yellow crystals. The yellow supernatant was decanted and the yellow solid was dried *in vacuo*, yielding compound **5**. Isolated yield: 55% yield (20.7 mg, 0.058 mmol). ^{1}H NMR ($CDCl_3$, 400 MHz, 23 °C) δ 8.04 (m, 2H), 7.60 (m, 1H), 7.49 (m, 2H), 2.00 (m, 3H), 1.87 (m, 6H), 1.67 (m, 6H), 1.15 (d, J_{PH} = 13 Hz, 9H) ppm. ^{13}C{^{1}H} NMR (CD_3CN, 98 MHz, 23 °C) δ 225.1 (d, J_{PC} = 50 Hz, C=O), 213.9 (d, J_{PC} = 46 Hz, C=O), 141.6 (d, J_{PC} = 35 Hz, Ar), 133.9 (Ar), 129.2 (d, J_{PC} = 10 Hz, Ar), 128.9 (Ar), 51.9 (d, J_{PC} = 28 Hz), 37.8 (d, J_{PC} = 4), 36.7, 33.5 (d, J_{PC} = 15 Hz), 29.0 (d, J_{PC} = 10 Hz), 28.2 ppm. ^{31}P{^{1}H} NMR ($CDCl_3$, 161 MHz, 23 °C) δ 37 ppm. ATR IR: 1665, 1629 cm^{-1} (C=O). DART HRMS (positive mode): Calculated for $C_{22}H_{30}O_2P$: 357.198344. Observed: 357.200195.

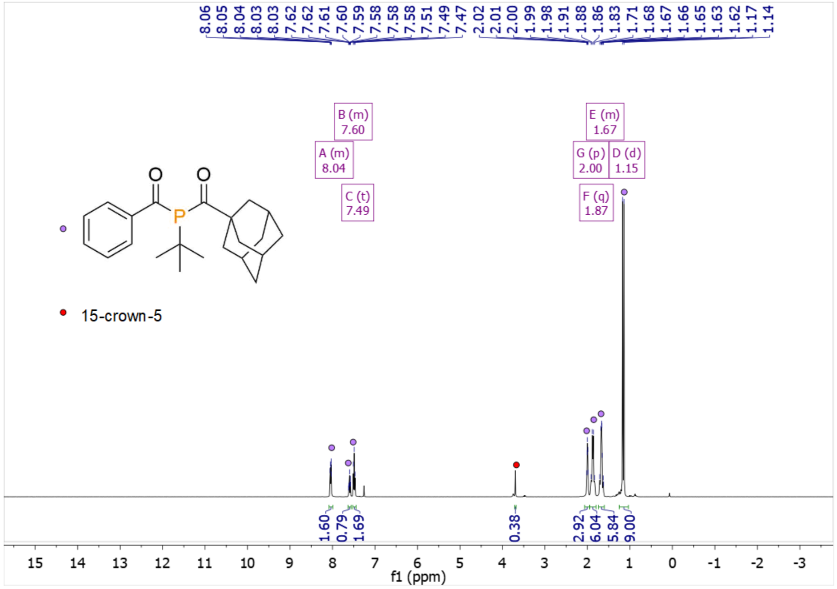

Figure S25: ^{1}H NMR spectrum of **5** in $CDCl_3$ at 25 °C, recorded at 400 MHz.

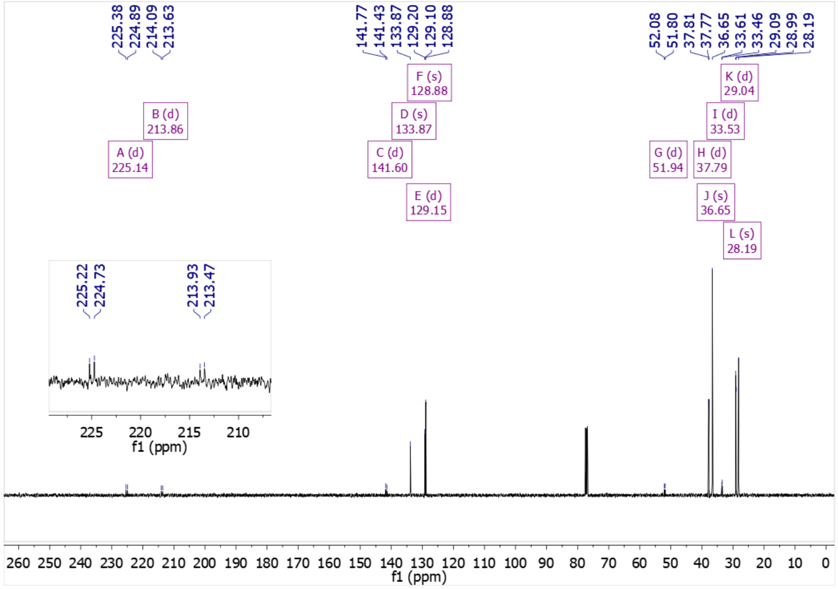

Figure S26: $^{13}C\{^{1}H\}$ NMR spectrum of **5** in $CDCl_3$ at 25 °C, recorded at 98 MHz. Downfield region of the $^{13}C\{^{1}H\}$ NMR spectrum of **5**.

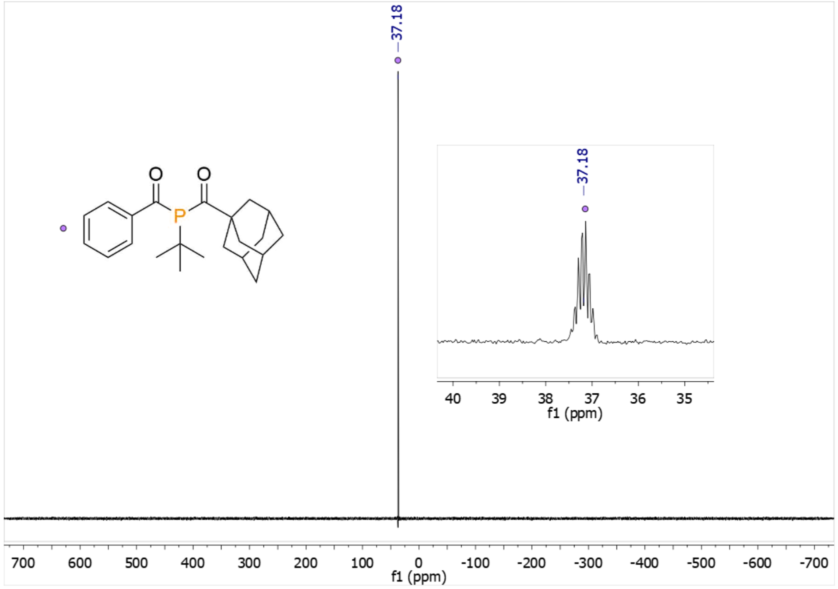

Figure S27: $^{31}P\{^{1}H\}$ NMR spectrum of **5** in $CDCl_3$ at 25 °C, recorded at 161 MHz. Insert: ^{31}P NMR spectrum of **5** in $CDCl_3$ at 25 °C, recorded at 161 MHz.

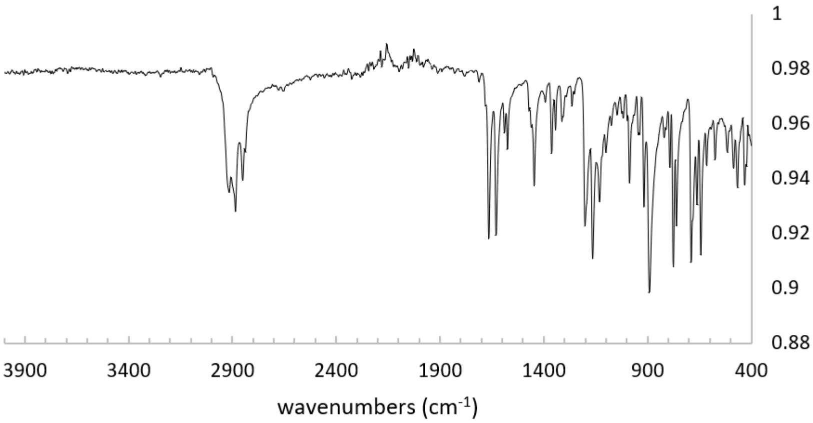

Figure S28: ATR IR spectrum of **5**.

S1.9 Competition experiments

Competition experiments to obtain a Hammett plot for the reaction between *t*-BuP**A** and *para*-substituted acyl chlorides were conducted in duplicate and using a modified literature procedure.[S11] In a representative experiment, *t*-BuP**A** (10.0 mg, 0.038 mmol,

1 equiv.) was dissolved in CH_2Cl_2 (0.40 mL measured by syringe). In a separate 20 mL scintillation vial, 3 equivalents of each acyl chlorides were combined and dissolved in CH_2Cl_2 (0.20 mL measured by syringe). The solution of *t*-BuP**A** was added to the acyl chloride mixture, and the reaction mixture was agitated by hand for 1 minute. $CDCl_3$ (5 drops) was added to the reaction mixture, which was then transferred to an NMR tube. Competition reactions were performed between *p*-H and *para*-CF_3, *para*-F, *para*-*t*-Bu and *para*-OMe. Ratios of products were determined by quantitative $^{31}P\{^1H\}$ NMR spectroscopy experiments, where d_1 was set to 35 seconds to ensure full relaxation of all ^{3}1P nuclei. This value was chosen based off measuring the T_1 of the ^{3}1P nuclei in the *para*-F and *para*-H derivatives (Fig. S29), which were found to be 6.7 and 7.2 s respectively. A d_1 of 35 s (T_1*5) was chosen to ensure relaxation of the ^{3}1P NMR resonances, and guarantee accuracy of the relative integrations to within 1%.[S12] The T_1 values for all ^{3}1P NMR nuclei were assumed to be 7 s based off this experiment. Representative NMR spectra for the competition experiments are shown below.

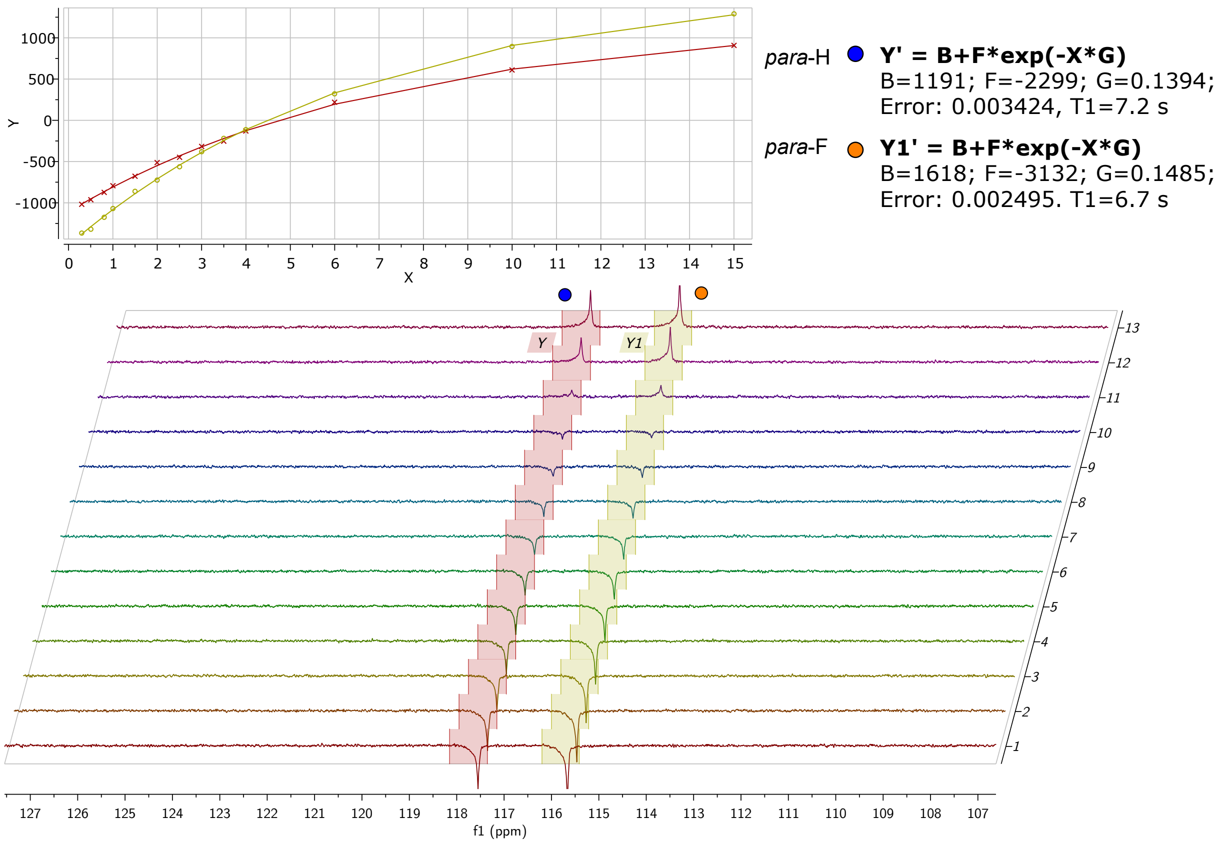

Figure S29: NMR spectra recorded during an inversion recovery experiment to measure the T_1 of two acylchlorophosphine derivatives at 25 °C, recorded at 161 MHz.

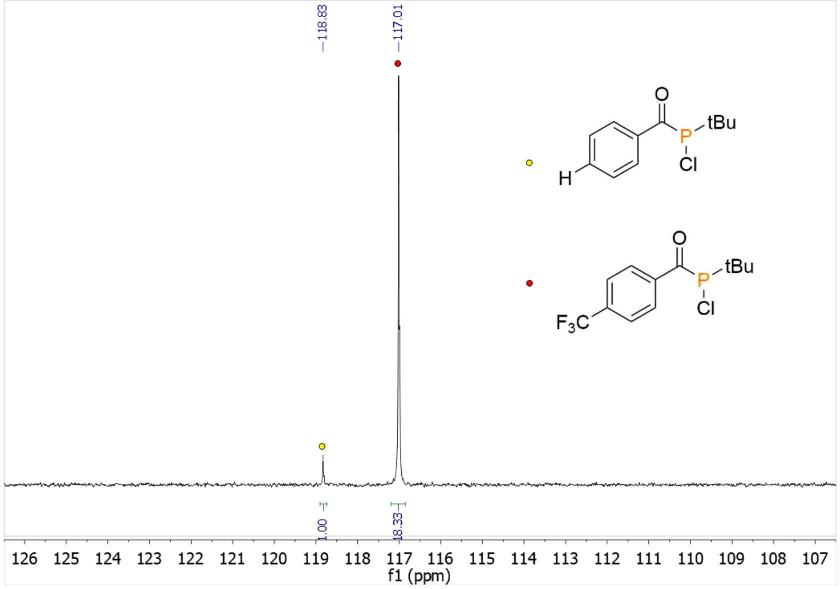

Figure S30: $^{31}P\{^{1}H\}$ NMR spectrum of reaction mixture containing **1** and *p*-CF_3-substituted variant in $CH_2Cl_2/CDCl_3$ at 25 °C, recorded at 161 MHz.

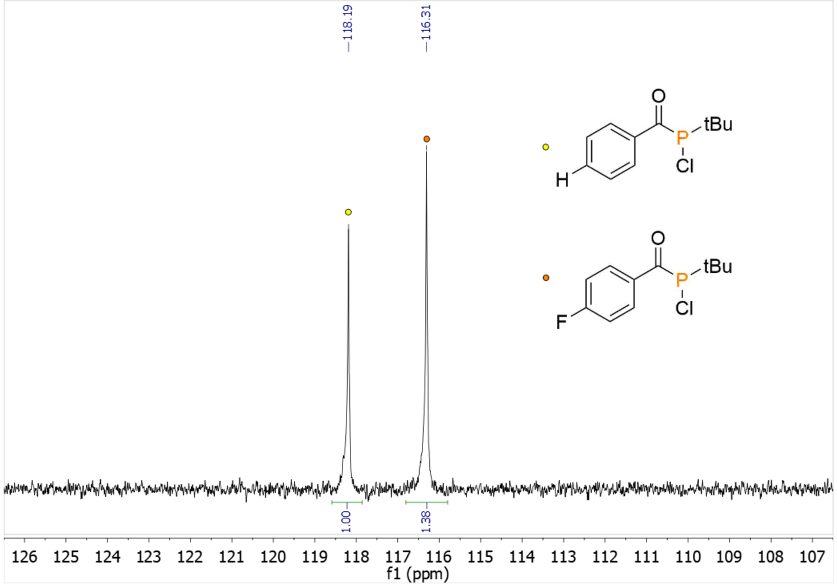

Figure S31: $^{31}P\{^{1}H\}$ NMR spectrum of reaction mixture containing **1** and *p*-F-substituted variant in $CH_2Cl_2/CDCl_3$ at 25 °C, recorded at 161 MHz.

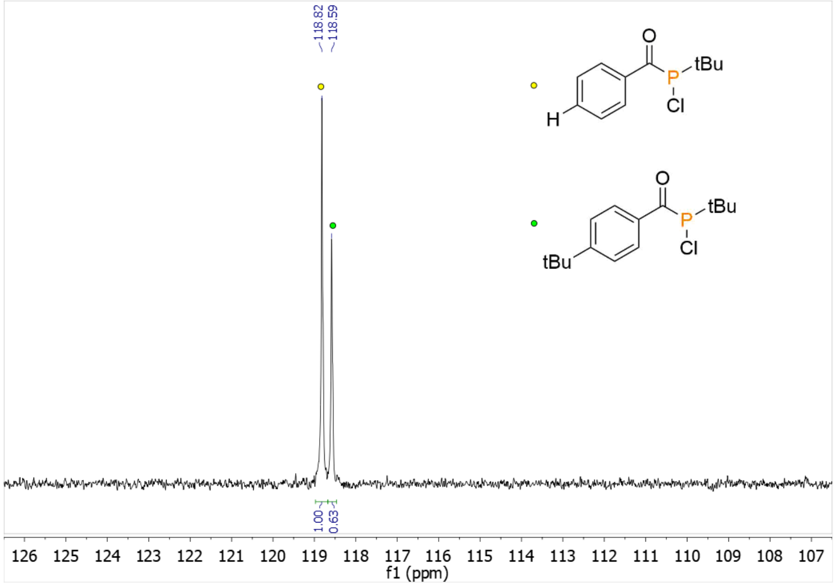

Figure S32: $^{31}P\{^1H\}$ NMR spectrum of reaction mixture containing **1** and *p*-(*t*-Bu)-substituted variant in $CH_2Cl_2/CDCl_3$ at 25 °C, recorded at 161 MHz.

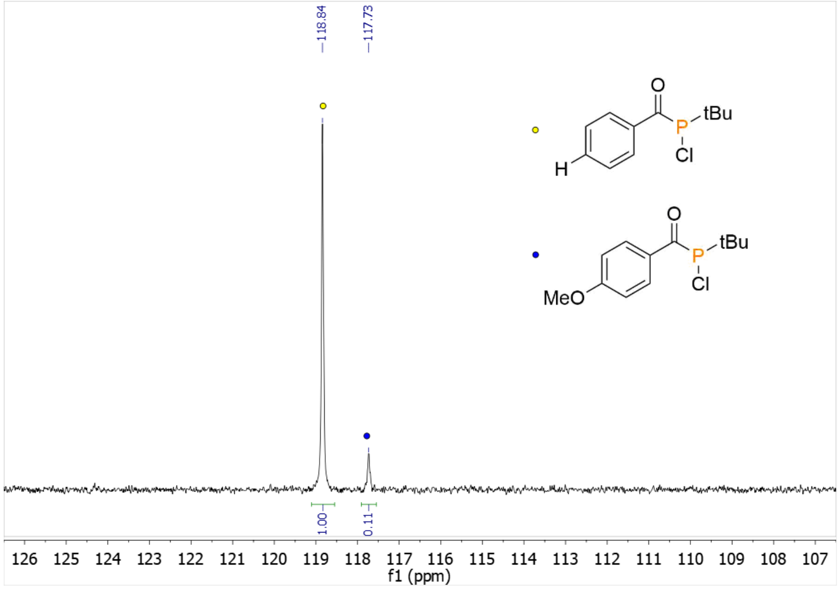

Figure S33: $^{31}P\{^1H\}$ NMR spectrum of reaction mixture containing **1** and *p*-OMe-substituted variant in $CH_2Cl_2/CDCl_3$ at 25 °C, recorded at 161 MHz.

S2 X-ray crystallographic studies

S2.1 General methods

Single crystals suitable for X-ray diffraction were transferred from the glovebox under Paratone oil onto a microscope slide. A crystal was selected under a microscope and mounted in hydrocarbon oil on a nylon loop. Low-temperature (100 K) data were collected on a Bruker-AXS X8 Kappa Duo diffractometer coupled to a Smart Apex2 CCD detector with Mo Kα radiation ($\lambda = 0.71073$ Å) with ϕ- and ω-scans. A semi-empirical absorption correction was applied to the diffraction data using SADABS.[S13,S14] The structure was solved by direct methods using SHELXT[S15,S16] and refined against F^2 on all data by full-matrix least squares with ShelXle.[S17] All non-hydrogen atoms were refined anisotropically. All hydrogen atoms were included in the model at geometrically calculated positions and refined using a riding model. The isotropic displacement parameters of all hydrogen atoms were fixed to 1.2 times the U_{eq} value of the atoms they are linked to (1.5 times for methyl groups).

S2.2 Compound 2

Single crystals of **2** were grown according to section S1.3. Compound **2** was solved in the space group $P2_1$/c. The model was first refined isotropically, then anisotropically. After calculating the positions of the hydrogen atoms using the HFIX command, the weighting scheme was updated and the model was judged to be complete.

Table S1: X-ray crystallographic information for **2**

CSD identification code	1882892
Reciprocal net code	X8_18132
Empirical formula	$C_{21}H_{28}Cl_3OPRu$
Formula weight	534.82 g/mol
Color / morphology	orange / block
Temperature	100(2) K
Wavelength	0.71073 Å
Crystal system	Monoclinic
Space group	$P2_1/c$
Unit cell dimensions	a = 15.7335(9) Å α = 90°<br>b = 7.0904(4) Å β = 105.282°<br>c = 20.8392(11) Å γ = 90°
Volume	2242.6(2) $Å^3$
Z	4
Density (calculated)	1.584 g/cm^3
Absorption coefficient	1.137 mm^{-1}
$F(000)$	1088
Crystal size	0.185 × 0.176 × 0.090 mm^3
Theta ranges for data collection	2.026 to 31.580°
Index ranges	−23<=h<=23, −10<=k<=10, −30<=l<=30
Reflections collected	173034
Independent reflections	7510 [R_{int} = 0.0543]
Completeness to θ = 25.242°	100.0%
Absorption correction	Semi-empirical from equivalents
Refinement method	Full-matrix least-squares on F^2
Data \restraints \parameters	7510 \0 \250
Goodness-of-fit on F^2	1.257
Final R indices [$I>2\sigma(I)$]	R_1 = 0.0482, wR_2 = 0.1047
R indices (all data)	R_1 = 0.0535, wR_2 = 0.1070
Extinction coefficient	n/a
Largest diff. peak and hole	1.647 and −0.963 $e{\cdot}Å^{-3}$

S2.3 Compound [Na(15-crown-5)]**4**

Single crystals of [Na(15-crown-5)]**4** were grown according to section S1.7. Compound **2** crystallizes in the space group $P2_1$/c. The model was first refined isotropically, then anisotropically. After calculating the positions of the hydrogen atoms using the HFIX command, the weighting scheme was updated and the model was judged to be complete. A small amount of disorder was observed in one of the 15-crown-5 molecules, but due to the low value of electron density assigned to these Q-peaks, the disorder was not treated.

Table S2: X-ray crystallographic information for [Na(15-crown-5)]**4**

CSD identification code	1882891
Reciprocal net code	X8_18100
Empirical formula	$C_{21}H_{34}NaO_6P$
Formula weight	436.44 g/mol
Color / morphology	yellow / block
Temperature	100(2) K
Wavelength	0.71073 Å
Crystal system	Monoclinic
Space group	$P2_1/c$
Unit cell dimensions	a = 18.5288(17) Å α = 90°<br>b = 14.5736(14) Å β = 113.995(2)°<br>c = 19.2314(18) Å γ = 90°
Volume	4744.3(8) $Å^3$
Z	8
Density (calculated)	1.222 g/cm^3
Absorption coefficient	0.166 mm^{-1}
$F(000)$	1872
Crystal size	0.277 × 0.192 × 0.169 mm^3
Theta ranges for data collection	1.203 to 27.977°
Index ranges	−24<=h<=24, −19<=k<=19, −25<=l<=25
Reflections collected	314225
Independent reflections	11401[R_{int} = 0.0647]
Completeness to θ = 25.242°	100.0%
Absorption correction	Semi-empirical from equivalents
Refinement method	Full-matrix least-squares on F^2
Data \restraints \parameters	11401 \0 \530
Goodness-of-fit on F^2	1.032
Final R indices [$I>2\sigma(I)$]	R_1 = 0.0406, wR_2 = 0.1050
R indices (all data)	R_1 = 0.0502, wR_2 = 0.1137
Extinction coefficient	n/a
Largest diff. peak and hole	1.175 and −0.415 $e{\cdot}Å^{-3}$

S2.4 Compound 5

Single crystals of **5** were grown according to section S1.8. Compound **5** crystallizes in the chiral space group $P2_1$. The single crystal that was selected for the X-ray diffraction study was identified as the (R) enantiomer; the Flack parameter was found to be zero within error, 0.062(116). The single crystal that was chosen showed non-merohedral twinning. A second domain was identified using CELL_NOW, and the two domains were integrated in APEX 2. The data was scaled using TWINABS. The structure was initially refined against the HKLF 4 reflection file, first isotropically then anisotropically. The positions of the hydrogen atoms were calculated using the HFIX command. The model was then refinement against the HKLF 5 reflection file. The weighting scheme was adjusted and the model was judged to be complete.

Table S3: X-ray crystallographic information for **5**

CSD identification code	1882890
Reciprocal net code	X8_18108_t5
Empirical formula	$C_{22}H_{29}O_2P$
Formula weight	356.42 g/mol
Color / morphology	yellow / plate
Temperature	100(2) K
Wavelength	0.71073 Å
Crystal system	Monoclinic
Space group	$P2_1$
Unit cell dimensions	a = 6.4762(6) Å α = 90°<br>b = 7.8291(7) Å β = 92.615(3)°<br>c = 18.9197(17) Å γ = 90°
Volume	958.28(15) $Å^3$
Z	2
Density (calculated)	1.235 g/cm^3
Absorption coefficient	0.156 mm^{-1}
$F(000)$	384
Crystal size	0.105 × 0.096 × 0.059 mm^3
Theta ranges for data collection	1.077 to 30.790°
Index ranges	−9<=h<=9, −11<=k<=11, −27<=l<=27
Reflections collected	5983
Independent reflections	5983
Completeness to θ = 25.242°	99.8%
Absorption correction	Semi-empirical from equivalents
Refinement method	Full-matrix least-squares on F^2
Data \restraints \parameters	5983 \1 \230
Goodness-of-fit on F^2	1.037
Final R indices [$I>2\sigma(I)$]	R_1 = 0.0432, wR_2 = 0.0840
R indices (all data)	R_1 = 0.0539, wR_2 = 0.0875
Extinction coefficient	n/a
Largest diff. peak and hole	0.244 and −0.293 $e\cdot Å^{-3}$

S3 Computational studies

S3.1 General procedures

All calculations were performed using ORCA 4.0.0.2[S18] using the C3DDB computational resource.[S19] Structures were viewed using Avogadro[S20] or IQmol.[S21] Calculations were performed using the B3LYP functional, and the def2-TZVP basis sets on all atoms, except for oxygen and chlorine atoms, for which the def2-TZVPP basis sets were used. The additional diffuse functions on chlorine and oxygen were necessary to locate various transition state geometries. Solvent effects were accounted for using the CPCM keyword, with toluene as the solvent. A typical input file is shown below:

```
%pal nprocs 16 end
% MaxCore 8000

! B3LYP D3 RIJCOSX def2-TZVP def2/J TightSCF Grid4 GridX4 FinalGrid5 Opt NumFreq
  CPCM(Toluene)

%basis newGTO Cl "def2-TZVPP" end
newGTO O "def2-TZVPP" end
end

* xyzfile 0 1 H.xyz
```

All optimized geometries were analyzed using a numerical frequencies calculation. Intermediate and transition state geometries were found to have zero and one imaginary

frequencies, respectively. An IRC calculation was performed on the imaginary frequency of the transition state geometries, which were shown to correspond to the intermediates connected by dotted lines in Fig. S34.

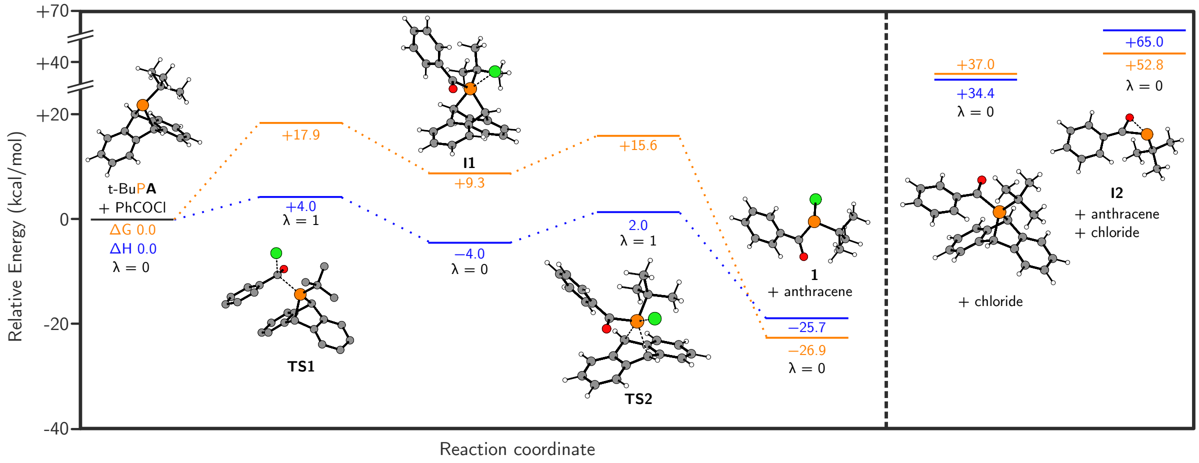

Figure S34: Calculated lowest energy pathway for the reaction of *t*-BuP**A** with benzoyl chloride to give **1**.

S3.2 Natural Bond Order (NBO) analysis

The initial structure of anion **4** was taken from the X-ray crystal structure, then optimized at the level of theory described in section S3.1. NBO analysis was carried out using NBO 6 within the ORCA program. The NRT keyword was specified to generate natural resonance structures of anion **4**. The lower contributing resonance structures (all below 2%) were considered negligible and not shown in the paper.

S3.3 Tables of optimized coordinates

Table S4: Optimized coordinates of benzoyl chloride.

Atom	x	y	z
C	-1.25651843402975	0.66767687999191	-0.15029317519807
C	-0.02532270122495	-1.54005010166325	-0.06509343477820
C	-1.25317068535527	-0.72337076085387	-0.02959696197080
C	-2.45903651133520	1.35958663815592	-0.12884214603841
C	-3.65797028627925	0.67184045769567	0.02690713910672
C	-3.65857730352662	-0.71522373334091	0.15226374892768
C	-2.46322623827209	-1.41384172050275	0.11814730099081
H	-2.45967001262229	2.43645884922731	-0.23170536204683
H	-4.59346377267914	1.21599459790519	0.05076429120214
H	-4.59115902718381	-1.24972990892217	0.27569872502758
H	-2.44849781046422	-2.49063461544018	0.21058557369193
H	-0.32576928343314	1.20163024259373	-0.26712388655228
O	0.03771307099480	-2.72382040511623	-0.14374100468130
Cl	1.53132899541092	-0.59999641973036	0.03867919231904

Table S5: Optimized coordinates of *t*-BuP**A**.

Atom	x	y	z
C	2.347010662	-1.345792949	-0.053006603
P	0.460661715	-1.513325123	-0.093260037

C	2.986699782	-0.066048024	-0.595905973
C	2.847066427	-2.535811116	-0.891688936
C	0.560817013	2.278584427	-1.328693663
H	0.538982142	2.33655062	-2.410235813
C	1.01858816	3.363978965	-0.571002398
H	1.347854455	4.265830197	-1.07142344
C	1.061185182	3.288876562	0.814892964
H	1.42377244	4.132017742	1.389232173
C	0.637915167	2.129261352	1.477601452
H	0.666005262	2.074977361	2.559216203
C	0.200260259	1.055999765	0.727189733
C	0.171501791	1.127233088	-0.675096636
C	-2.931162403	-0.471464629	-1.428080694
H	-2.942956848	-0.429918431	-2.510382609
C	-4.119851487	-0.633948971	-0.711045874
H	-5.05942188	-0.715324213	-1.242847644
C	-4.10662668	-0.695050031	0.67834669
H	-5.036018545	-0.823586949	1.21862439
C	-2.904325792	-0.594989198	1.384249778
H	-2.895825049	-0.648909979	2.466031396
C	-1.728944339	-0.428516291	0.675265532
C	-1.742423426	-0.368043959	-0.729228147
C	-0.323660278	-0.186409354	-1.247152368
H	-0.202036594	-0.316499904	-2.318205519
C	-0.300685926	-0.298949237	1.181814229
H	-0.162044705	-0.531463605	2.233003534
C	2.753036156	-1.548067792	1.414079678
H	2.286594487	-2.437470784	1.846095422
H	2.476352575	-0.686033212	2.022971931
H	3.837294461	-1.675241442	1.481330293
H	2.687217959	0.128577728	-1.62609448
H	4.074711668	-0.187055791	-0.583715827
H	2.741644167	0.80946999	-0.000288905
H	2.53711487	-2.444254522	-1.935765063
H	2.468172564	-3.485683912	-0.510661977
H	3.94011359	-2.56880038	-0.869606792

Table S6: Optimized coordinates of TS1.

Atom	x	y	z
C	2.599916715	-1.475219011	0.283545114

P	0.762842361	-1.541150673	-0.070243696
C	3.266675367	-0.098110148	0.20639838
C	3.254384179	-2.399809687	-0.758078076
C	1.134516497	1.981059877	-1.758144829
H	1.349233452	1.886625823	-2.815435892
C	1.409386866	3.173654977	-1.081245498
H	1.837113338	4.008740648	-1.621174538
C	1.138590901	3.295750677	0.276763517
H	1.356794816	4.225325697	0.786651105
C	0.587169201	2.2277675	0.992219113
H	0.381185255	2.323460073	2.051007616
C	0.328307241	1.046221788	0.325252263
C	0.600205027	0.923910635	-1.047483058
C	-2.317309387	-0.757979901	-2.19603283
H	-2.110914788	-0.866170074	-3.252699806
C	-3.626441021	-0.820782067	-1.71803488
H	-4.442131256	-0.970546804	-2.413345207
C	-3.892160099	-0.714877283	-0.356734358
H	-4.91311272	-0.77954653	-0.002822943
C	-2.854562919	-0.538601375	0.560583339
H	-3.060584793	-0.470945622	1.621037475
C	-1.559269369	-0.46387437	0.085482354
C	-1.29151428	-0.579297785	-1.287243773
C	0.206383141	-0.459740819	-1.536688604
H	0.544934231	-0.736681806	-2.529129307
C	-0.261776812	-0.248750205	0.857135092
H	-0.333701678	-0.347409308	1.93453582
C	2.765444612	-2.03719747	1.706292925
H	2.322896499	-3.025867931	1.810059017
H	2.321034403	-1.368380729	2.44617002
H	3.833001702	-2.116845867	1.92686605
H	3.196590319	0.340736096	-0.786375999
H	4.326231682	-0.236296756	0.4396109
H	2.854123136	0.607355345	0.923522385
H	3.130859723	-2.003128639	-1.768550883
H	2.850315329	-3.409039904	-0.718252329
H	4.326303362	-2.452894735	-0.552040319
C	-0.458733117	-3.452790397	0.401966431
O	-0.940180718	-3.285492439	1.479511289
C	-1.149224598	-3.786569694	-0.874498456
C	-2.521116934	-4.010272463	-0.811205602
C	-0.483490333	-3.864030861	-2.09518417
C	-1.188945214	-4.160003345	-3.248380337
C	-2.564240274	-4.387981559	-3.188650349

C	-3.225487347	-4.314722997	-1.970903717
H	0.585542857	-3.704524526	-2.132122794
H	-0.669559529	-4.221371346	-4.195991736
H	-3.11338347	-4.621441377	-4.091914996
H	-4.293249832	-4.484218216	-1.918995807
H	-3.026138888	-3.93488895	0.141053156
Cl	1.11856081	-4.929770067	0.47256143

Table S7: Optimized coordinates of I1.

Atom	x	y	z
C	1.859148809	-1.151321933	-0.694629802
P	0.064772715	-1.111349473	-0.022257412
C	1.949948506	-0.371008588	-2.013607436
C	2.34328353	-2.584221168	-0.96768686
C	0.354601963	2.678923286	-1.011648776
H	0.209799394	2.835052846	-2.07361772
C	1.019574068	3.633421819	-0.240783955
H	1.386744455	4.537604418	-0.709211356
C	1.215913033	3.433677007	1.123481787
H	1.73604372	4.182565521	1.706763114
C	0.747996522	2.275509224	1.745487652
H	0.905534355	2.115009662	2.804476495
C	0.085557924	1.332439752	0.978485725
C	-0.109161675	1.527531436	-0.396005672
C	-3.448880764	0.282364837	-0.834904746
H	-3.597056699	0.41068221	-1.900144757
C	-4.541872948	0.14023777	0.021829966
H	-5.545665619	0.164969103	-0.382675586
C	-4.353898996	-0.039631733	1.389388112
H	-5.212841706	-0.153413101	2.038209325
C	-3.06903031	-0.089925183	1.931023502
H	-2.921699714	-0.258175222	2.990125354
C	-1.988875565	0.061569989	1.081767738
C	-2.174807711	0.253056031	-0.295644362
C	-0.838572935	0.347163262	-0.993229209
H	-0.873565177	0.320508916	-2.076598222
C	-0.51212998	0.015451769	1.413563628
H	-0.248973494	-0.341383996	2.399020285
C	2.771107064	-0.494139445	0.353598714

H	2.687910049	-0.992210984	1.317304256
H	2.554582149	0.564720094	0.476594738
H	3.802203116	-0.590305166	0.005644543
H	1.301080541	-0.784678933	-2.783598134
H	2.977067034	-0.459302678	-2.373813743
H	1.733682629	0.68538335	-1.891361796
H	1.788517241	-3.056922189	-1.774321788
H	2.290213066	-3.210510707	-0.084004863
H	3.387009773	-2.511805798	-1.282560418
C	-1.110534351	-2.569835823	-0.438731793
O	-2.041695827	-2.819889683	0.274638605
C	-0.798285904	-3.357407644	-1.657439648
C	-0.643479172	-2.748400895	-2.901209305
C	-0.678502785	-4.742206369	-1.533709542
C	-0.372024815	-5.507833857	-2.649629281
C	-0.2175032	-4.899748535	-3.892744957
C	-0.366720959	-3.522537318	-4.020260654
H	-0.798012061	-5.199006079	-0.560698258
H	-0.254666185	-6.579120588	-2.550914089
H	0.015958076	-5.500361985	-4.762379355
H	-0.263636819	-3.050790079	-4.988609501
H	-0.761948357	-1.679510651	-3.003977087
Cl	0.721001387	-2.78642094	1.790845115

Table S8: Optimized coordinates of TS2.

Atom	x	y	z
C	2.509070843	-1.286282429	-0.243439502
P	0.652049339	-1.196377182	-0.717188512
C	3.179682146	-0.02808585	-0.819941033
C	3.174446627	-2.551523949	-0.797901619
C	0.436294287	2.660885982	-0.971144602
H	0.25283833	2.91288462	-2.008464784
C	1.196338977	3.494824133	-0.166862475
H	1.598676876	4.415235532	-0.569926907
C	1.451343838	3.147184809	1.165763027
H	2.047598311	3.80444543	1.786109069
C	0.952549917	1.968709334	1.695511024
H	1.163677929	1.699502043	2.723390695
C	0.16769194	1.122395304	0.901448352

C	-0.095051042	1.484034736	-0.439396191
C	-3.12268621	-0.556719629	-1.124712314
H	-3.30585665	-0.301113145	-2.160943642
C	-4.060752917	-1.265300362	-0.397209304
H	-4.997495505	-1.552244491	-0.857078007
C	-3.794808413	-1.632350117	0.92948371
H	-4.532194158	-2.197074225	1.486017651
C	-2.598939912	-1.285965274	1.53222672
H	-2.395538682	-1.580048503	2.554705954
C	-1.651709916	-0.534629993	0.821528501
C	-1.923080044	-0.170247512	-0.51814549
C	-0.804304676	0.461872252	-1.192689687
H	-0.876238655	0.587890391	-2.265446559
C	-0.325375154	-0.186474551	1.285435767
H	-0.028015357	-0.552067278	2.261333317
C	2.674020204	-1.270497785	1.280411892
H	2.187462486	-2.115176605	1.76079819
H	2.310047483	-0.348168249	1.723527232
H	3.742975906	-1.342678215	1.496800064
H	3.14578642	-0.011427675	-1.907016199
H	4.228112808	-0.030404267	-0.510655866
H	2.72278948	0.885989286	-0.441972654
H	3.05558188	-2.642371253	-1.874374611
H	2.781923502	-3.450871739	-0.32363265
H	4.244515748	-2.499037608	-0.57798723
Cl	1.003977179	-1.387611104	-2.873267598
C	-0.205609783	-2.908700649	-0.634737992
O	-1.034761273	-3.14871843	-1.476600808
C	0.154156017	-3.911409568	0.401884628
C	0.40950921	-5.212290877	-0.044511461
C	0.215649118	-3.625412589	1.764264053
C	0.534209965	-4.62834965	2.671300387
C	0.816402289	-5.91354218	2.221919702
C	0.755625511	-6.203439149	0.861487102
H	-0.014505174	-2.6347174	2.119667423
H	0.561420026	-4.404527675	3.729820634
H	1.077682607	-6.689537728	2.929841253
H	0.971558414	-7.203376054	0.508103318
H	0.349017908	-5.429302913	-1.102491967

Table S9: Optimized coordinates of PhC(O)P(Cl)(t-Bu).

Atom	x	y	z
C	-1.210203941	0.662841332	0.31385418
C	-0.113399379	-1.550090133	0.829511194
C	-1.289421038	-0.724797837	0.453196159
C	-2.344595955	1.396259734	-0.005201345
C	-3.558293248	0.747248904	-0.209486248
C	-3.641880346	-0.637062073	-0.079222901
C	-2.515260014	-1.369569968	0.260920913
H	-2.281245033	2.472514527	-0.098056775
H	-4.439506437	1.319906611	-0.469477652
H	-4.585718628	-1.141356067	-0.24138186
H	-2.565969815	-2.444009961	0.374676285
H	-0.270486353	1.174127976	0.473370991
O	-0.238450883	-2.606048956	1.417125709
P	1.624395134	-0.859887042	0.500950274
C	2.694372226	-2.402390451	0.416113059
C	4.056186212	-1.941991595	-0.127648141
C	2.135018725	-3.524633009	-0.458404747
H	4.475478676	-1.129901103	0.469981278
H	3.985745462	-1.607421369	-1.16249097
H	4.752593478	-2.783436369	-0.090328791
H	1.986363509	-3.188605605	-1.485038177
H	1.191512366	-3.903821771	-0.071763691
H	2.851700337	-4.350565454	-0.476910897
C	2.866646358	-2.868903558	1.872811343
H	3.238491359	-2.063552725	2.510699047
H	3.603525414	-3.676158262	1.894852215
H	1.933049185	-3.242184561	2.287321779
Cl	1.40833872	-0.378483531	-1.530250393

Table S10: Optimized coordinates of anthracene.

Atom	x	y	z
C	3.645438694	-0.711093759	-0.00007925
C	3.645436813	0.711087202	-0.00007913
C	2.469524606	1.402729777	-0.00005782
C	1.218160323	0.719912107	-0.00005989
C	2.469518762	-1.402724433	-0.00005730
C	1.218156203	-0.71990359	-0.00006021

C	-8.40325E-07	1.399989365	0.00000057
C	-1.218157432	0.719906713	0.00006051
C	-2.18318E-06	-1.399985988	0.00000111
C	-1.21815794	-0.719905071	0.00006121
C	-2.469520596	1.402727054	0.00005743
C	-3.645438204	0.711089727	0.00007925
C	-3.64543518	-0.711092561	0.00007936
C	-2.469517264	-1.402730518	0.00005579
H	4.589235141	-1.241458831	0.00009715
H	4.589238328	1.241444959	0.00009824
H	2.46640629	2.486214857	0.00001372
H	2.466398715	-2.486209875	0.00001700
H	-5.67006E-06	2.48422954	0.00000185
H	1.30143E-06	-2.484225677	0.00000221
H	-2.466404816	2.486213515	-0.00001555
H	-4.589236966	1.241450355	-0.00009836
H	-4.589235826	-1.241451266	-0.00010056
H	-2.466399258	-2.486215601	-0.00001632

Table S11: Optimized coordinates of [PhC(O)P(t-Bu)**A**]$^+$.

Atom	x	y	z
C	2.530824285	-1.318610899	-0.32983776
P	0.681997847	-1.267548948	-0.297164943
C	3.119685527	0.013090943	-0.813998749
C	2.929948204	-2.449264591	-1.29382882
C	0.506830205	2.40218641	-1.406033997
H	0.471838164	2.494310795	-2.483872928
C	0.903086975	3.479863811	-0.611494659
H	1.169875725	4.418049815	-1.079697931
C	0.965177734	3.3589689	0.773285706
H	1.280253588	4.20387443	1.371192638
C	0.627981578	2.158065122	1.401950002
H	0.681257758	2.063933898	2.47878285
C	0.227086712	1.095222853	0.614641425
C	0.170778085	1.215970335	-0.781964993
C	-2.825557754	-0.661158318	-1.554546873
H	-2.861079792	-0.582842489	-2.633177258
C	-3.967004886	-0.995224328	-0.825305239
H	-4.900123792	-1.16898458	-1.344620466

C	-3.917450414	-1.116002822	0.560678571
H	-4.81257828	-1.383269676	1.106549628
C	-2.726099016	-0.903896168	1.255944015
H	-2.68618427	-1.011507334	2.331894921
C	-1.598523538	-0.558078669	0.534451557
C	-1.648857863	-0.439315692	-0.86386677
C	-0.272193319	-0.095112918	-1.415775577
H	-0.146291514	-0.175154466	-2.488828006
C	-0.181468067	-0.306050667	1.041153018
H	0.01870119	-0.589674121	2.067525124
C	3.004993608	-1.627334441	1.100484766
H	2.595350809	-2.562718804	1.479812373
H	2.744688473	-0.823662874	1.789745071
H	4.092999614	-1.71448739	1.081493522
H	2.780482666	0.269438097	-1.81724633
H	4.205006272	-0.101198388	-0.845316169
H	2.88491769	0.83692315	-0.144012172
H	2.573619342	-2.266516687	-2.308773085
H	2.568843951	-3.423293701	-0.963408956
H	4.019615968	-2.494313788	-1.329798563
C	0.019391004	-3.039997113	-0.103057231
O	0.159726042	-3.446940302	1.027774359
C	-0.612531265	-3.755857699	-1.206475181
C	-0.497616655	-3.313300518	-2.527421939
C	-1.349619508	-4.912964843	-0.917839494
C	-1.970452492	-5.60447756	-1.942758737
C	-1.849924837	-5.158132846	-3.258430292
C	-1.107946305	-4.017323624	-3.552199655
H	-1.432086858	-5.244433044	0.108030423
H	-2.549242679	-6.491433798	-1.72304871
H	-2.337019404	-5.702351232	-4.057114079
H	-1.010493982	-3.679453201	-4.574912035
H	0.079370876	-2.430586154	-2.764371371

Table S12: Optimized coordinates of [PhC(O)P(t-Bu)]$^+$.

Atom	x	y	z
C	1.803322213	-1.050354316	-0.999080865
P	0.009871363	-1.383150069	-1.341314641
C	2.408101031	-1.810683705	0.177285407

C	1.791707614	0.471390131	-0.739380608
C	2.550766657	-1.366033431	-2.304663413
H	2.119379378	-0.841534019	-3.15948505
H	2.547814089	-2.436098902	-2.51635835
H	3.588971726	-1.043588587	-2.202463782
H	1.926068583	-1.55530796	1.121804228
H	3.463564721	-1.544884012	0.262381766
H	2.349455165	-2.890079794	0.034188701
H	1.298312971	0.721309829	0.20132457
H	1.312338613	1.02956557	-1.547579701
H	2.826896639	0.814161631	-0.678726218
C	-0.532934906	-2.212676154	0.131565221
O	-0.249857425	-3.118742233	-0.719887835
C	-1.013087212	-2.288259744	1.445340404
C	-1.241660659	-1.089704415	2.151300536
C	-1.260767693	-3.54237973	2.045850174
C	-1.732174251	-3.582275051	3.339560473
C	-1.95740334	-2.389757515	4.034862083
C	-1.714372773	-1.147805664	3.446274634
H	-1.079240136	-4.450473314	1.487204835
H	-1.929350587	-4.531592503	3.817733558
H	-2.329044155	-2.43172807	5.050499261
H	-1.897358003	-0.23893315	4.001874172
H	-1.048979621	-0.140344823	1.66961044

Table S13: Optimized coordinates of $[PhC(O)P(t\text{-}Bu)]^-$.

Atom	x	y	z
P	-4.500538	9.969281	1.803432
O	-1.969469	9.113771	2.397862
C	-2.724825	9.894513	1.761794
C	-2.047757	10.937555	0.897872
C	-0.785554	11.404744	1.28044
C	-0.115463	12.360406	0.526268
C	-0.691014	12.861964	-0.638508
C	-1.941195	12.396776	-1.038353
C	-2.61244	11.446112	-0.277203
C	-4.443444	8.736186	4.37157
C	-6.531983	8.547984	3.015939
C	-4.533196	7.186721	2.398107

C	-4.997701	8.539002	2.954703
H	-3.354596	8.758438	4.3568
H	-4.766566	7.915779	5.024365
H	-4.805346	9.671314	4.805672
H	-6.972098	8.399344	2.026369
H	-6.910169	9.493542	3.413441
H	-6.887277	7.742548	3.66762
H	-4.949833	7.009934	1.403699
H	-4.866303	6.37112	3.051924
H	-3.446682	7.151044	2.32955
H	-0.337915	11.006192	2.181362
H	-0.167241	13.600129	-1.233102
H	0.857638	12.713854	0.845897
H	-2.391317	12.767747	-1.951157
H	-3.575175	11.076131	-0.604734

References

(S1) Pangborn, A. B.; Giardello, M. A.; Grubbs, R. H.; Rosen, R. K.; Timmers, F. J. Safe and Convenient Procedure for Solvent Purification. *Organometallics* **1996**, *15*, 1518–1520.

(S2) Williams, D. B. G.; Lawton, M. Drying of Organic Solvents: Quantitative Evaluation of the Efficiency of Several Desiccants. *J. Org. Chem.* **2010**, *75*, 8351–8354.

(S3) Velian, A.; Cummins, C. C. Facile Synthesis of Dibenzo-7λ^3-phosphanorbornadiene Derivatives Using Magnesium Anthracene. *J. Am. Chem. Soc,* **2012**, *134*, 13978–13981.

(S4) Freeman, P. K.; Hutchinson, L. L. Magnesium anthracene dianion. *J. Org. Chem.* **1983**, *48*, 879–881.

(S5) Brown, L.; Koreeda, M. Benzoyl trifluoromethanesulfonate. A mild reagent for the benzoylation of sterically hindered hydroxyls. *J. Org. Chem.* **1984**, *49*, 3875–3880.

(S6) Connelly, N. G.; Geiger, W. E. Chemical Redox Agents for Organometallic Chemistry. *Chem. Rev.* **1996**, *96*, 877–910.

(S7) Jayaraman, A.; Sterenberg, B. T. Phosphorus–Carbon Bond Forming Reactions of Diphenylphosphenium and Diphenylphosphine Triflate Complexes of Tungsten. *Organometallics* **2016**, *35*, 2367–2377.

(S8) Jayaraman, A.; Nilewar, S.; Jacob, T. V.; Sterenberg, B. T. Sequential Electrophilic Substitution Reactions of Tungsten-Coordinated Phosphenium Ions and Phosphine Triflates. *ACS Omega* **2017**, *2*, 7849–7861.

(S9) Nilewar, S.; Jayaraman, A.; Sterenberg, B. T. Alkyne Insertion into PC(sp^2) Bonds as a Route to Fused Phospholes: Transition-Metal-Like Reactivity at Phosphorus. *Organometallics* **2018**, *0*, null, 10.1021/acs.organomet.8b00718.

(S10) Dankowski, M.; Praefcke, K. Organische Photochemie XXXII: Beitrag über Photoreaktionen von Aroyl-diphenylphosphinen in Lösung. *Phosphorus Sulfur Relat. Elem.* **1980**, *8*, 105–108.

(S11) Mullins, R. J.; Vedernikov, A.; Viswanathan, R. Competition Experiments as a Means of Evaluating Linear Free Energy Relationships. An Experiment for the Advanced Undergraduate Organic Chemistry Lab. *J. Chem. Educ.* **2004**, *81*, 1357.

(S12) Saito, T.; Nakaie, S.; Kinoshita, M.; Ihara, T.; Kinugasa, S.; Nomura, A.; Maeda, T. Practical guide for accurate quantitative solution state NMR analysis. *Metrologia* **2004**, *41*, 213–218.

(S13) Bruker (2001). SADABS. Bruker AXS Inc., Madison, Wisconsin, USA.

(S14) Krause, L.; Herbst-Irmer, R.; Sheldrick, G. M.; Stalke, D. Comparison of silver and molybdenum microfocus X-ray sources for single-crystal structure determination. *J. Appl. Crystallogr.* **2015**, *48*, 3–10.

(S15) Sheldrick, G. M. *SHELXT* Integrated space-group and crystal-structure determination. *Acta Crystallogr., Sect. A: Found. Adv.* **2015**, *71*, 3–8.

(S16) Sheldrick, G. M. A short history of *SHELX*. *Acta Crystallogr., Sect. A: Found. Crystallogr.* **2008**, *64*, 112–122.

(S17) Hübschle, C. B.; Sheldrick, G. M.; Dittrich, B. *ShelXle* : a Qt graphical user interface for *SHELXL*. *J. Appl. Crystallogr.* **2011**, *44*, 1281–1284.

(S18) Neese, F. The ORCA program system. *Wiley Interdiscip. Rev.: Comput. Mol. Sci.* **2012**, *2*, 73–78.

(S19) Massachusetts Green High Performance Computing Center, https://www.mghpcc.org/.

(S20) Hanwell, M. D.; Curtis, D. E.; Lonie, D. C.; Vandermeersch, T.; Zurek, E.; Hutchison, G. R. Avogadro: an advanced semantic chemical editor, visualization, and analysis platform. *J. Cheminf.* **2012**, *4*, 17.

(S21) IQmol program for molecular visulization, http://iqmol.org/.
